# Outdoor Radon Dose Rate in Canada’s Arctic amid Climate Change

**DOI:** 10.1021/acs.est.4c02723

**Published:** 2024-06-22

**Authors:** Chuanlei Liu, Jing Chen, Weihua Zhang, Kurt Ungar

**Affiliations:** † Radiation Protection Bureau of Health Canada, 775 Brookfield Rd, Ottawa, Ontario K1A 1C1, Canada

**Keywords:** Fixed Point Surveillance network, soil gas emission, long-term trend, long-distance transport, temperature, precipitation, health risk, active layer

## Abstract

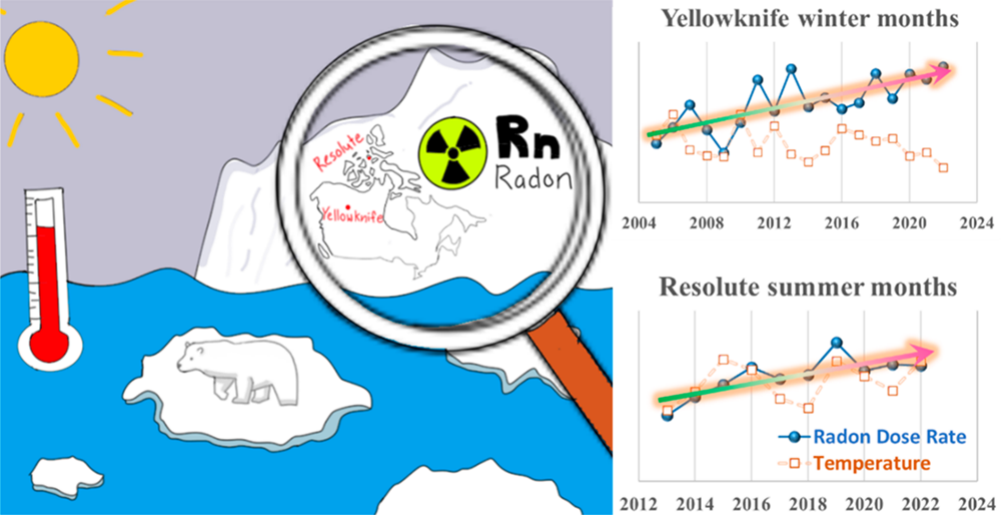

Decades of radiation monitoring data were analyzed to estimate outdoor Radon Dose Rates (RnDRs) and evaluate climate change impacts in Canada’s Arctic Regions (Resolute and Yellowknife). This study shows that the RnDR involves dynamic sources and complex environmental factors and processes. Its seasonality and long-term trends are significantly impacted by temperatures and soil-and-above water contents. From 2005 to 2022, Yellowknife’s RnDR increased by +0.35 ± 0.06 nGy/h per decade, with the fastest increases occurring in cold months (October to March). The rise is largely attributable to water condition changes over time in these months, which also caused enhanced soil gas emissions and likely higher indoor radon concentrations. In Resolute, the RnDR increased between 2013 and 2022 at +0.62 ± 0.19 nGy/h (or 16% relatively) per decade in summer months, with a positive temperature relationship of +0.12 nGy/h per °C. This work also demonstrates the relevance of local climate and terrain features (e.g., typical active layer depth, precipitation amount/pattern, and ground vegetation cover) in researching climate change implications. Such research can also benefit from using supporting monitoring data, which prove effective and scientifically significant. From the perspective of external exposure to outdoor radon, the observed climate change effects pose a low health risk.

## Abstract 2

This work demonstrated the climate change impacts on radon exposure in Canada’s Arctic and concluded low associated health risk from external radon exposure outdoors.

## 1 Introduction

Radon (^222^Rn), a progeny of uranium-238, occurs naturally in various environments. As entering an indoor environment, it can accumulate over time, resulting in elevated levels that can harm health.^1^ Studies indicate that about 3% to 14% of lung cancer is attributed to long-term exposure to indoor radon.^2,3^ Outdoors, it typically poses little health risks to the public due to its low concentration. It is more commonly used as an atmospheric tracer, a precursor to natural phenomena, and a proxy for indoor radon potential.^4−7^

Radon concentrations in a given region depend largely on local uranium reservoirs, geological features, and meteorological conditions, and they fluctuate on a daily, seasonal, and annual basis. Canada is abundant in uranium, with the majority being found in permafrost regions,^8^ which are also identified as high risk areas for radon exposure^9^ under a warming climate. Concerns about radon exposure are growing in recent years, particularly regarding the impact of climate change in areas that are experiencing rising temperatures, degrading permafrost, or shifting precipitation patterns.^8,10−12^

Research on the impact of climate change on radon exposure is lacking because it typically requires decades of simultaneous data on radon and various environmental conditions to achieve reliable results of statistical significance. Fortunately, Health Canada’s Fixed-Point Surveillance (FPS) network^13^ has collected decades of radiation monitoring data that can be leveraged. In this article, we used these data to study the outdoor Radon Dose Rates (RnDRs) in two Arctic regions (Yellowknife and Resolute), covering seasonality, long-term trends, and relationships with meteorological conditions. Unlike radon concentration measurements, RnDR is a measure of exposure to γ rays of short-lived radon daughters and was estimated using gamma spectrometry analysis of the ^214^Bi 1.765 MeV signals.

This work addressed public concerns from both health and climate change perspectives with two objectives. The first is to understand various radiation sources and complex environmental factors and processes impacting RnDRs. This also allows for establishing historical baselines for assessing future RnDR changes possibly caused by climate change or increased human activity in the Arctic. The second objective is to quantify long-term RnDR trends and determine the climatic impacts, particularly with regard to the air temperature and precipitation, two key indicators of climate change. Moreover, this work used three supporting radiation monitoring data sets to facilitate exploration of environmental effects. This approach proved to be relevant and highly effective in understanding various environmental phenomena and processes related to this research, including insights into long-term trends of indoor radon concentrations^14^ and soil gas emissions^15,16^ in Canada’s Arctic. More understanding was also gained by contrasting the data from two stations with different climatic and geological conditions.

## 2 Materials and Methods

### 2.1 The Fixed-Point Surveillance Network

The FPS network, established in 2002, was designed to monitor environmental γ radiation and assess health risks arising from both natural and anthropogenic radiation sources in Canada. To date, over 80 stations have been deployed near nuclear facilities, ports, and major cities in Canada. Each station employs a 7.62 cm × 7.62 cm sodium iodide spectrometer (RS250 detector) for real-time radiation detections and dose measurements.^17,18^

**Figure 1:**
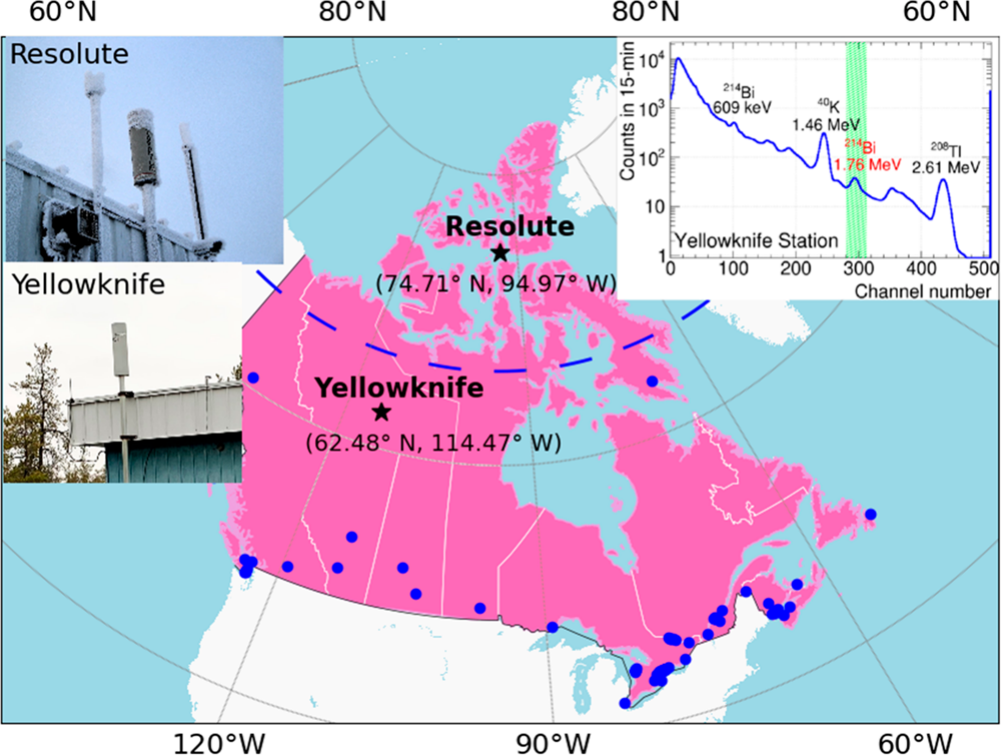
A map of the FPS network. Yellowknife and Resolute stations are highlighted as black stars, while all other stations are displayed as blue dots. The Arctic circle at 66°33′N is displayed as a dashed blue line. Image insets: the left side shows the Resolute and Yellowknife RS250 detectors, while the right side shows a typical 15 min energy spectrum (monthly averaged, and the channel number on the *x*-axis represents the energy deposited in the detector) collected from the Yellowknife station. The green hatched region in this inset indicates the energy/channel window that was used to count radon detections.

Figure 1 maps the FPS network, with Resolute (74.71° N, 94.97° W, Nunavut) and Yellowknife (62.48° N, 114.47° W, Northwest Territories/NWT) stations highlighted by black stars. Both stations are situated within Canada’s Arctic/Subarctic region but have distinct climate and geographical conditions.

Yellowknife falls within a discontinuous permafrost zone.^19^ The active-layer thickness in the region exhibits large spatial variability, ranging from tens to over 100 cm.^20−22^ The station surroundings consist of rock outcrops and muskegs in poorly drained and low-relief terrain. Resolute station, situated on Cornwallis Island in Nunavut, lies within a continuous permafrost zone. During July, the active layer near the station site can extend to a depth of 0.7–0.8 m.^23^ The area exhibits a polar desert profile, and plant life is sporadic and uncommon.

### 2.2 Outdoor Radon Dose Rate Measurements

As a dosimetry system, the FPS network provides health risk assessments for environmental radiation using gamma spectrometry.^17,18,24^ An example spectrum from the Yellowknife station (see the right-side inset of Figure 1) shows the typical gamma peaks originating from natural radiation sources.

Following geoscience recommendations,^25,26^ RnDRs in FPS were estimated using a conventional spectral window centered at the ^214^Bi 1.765 MeV position and approximately 0.2 MeV wide (hatched region in the right-side inset of Figure 1). To reduce noise arising from the Compton continuum of higher energy gammas, a stripping method^25−27^ was applied. The resulting net count rate was converted into the radionuclide air kerma rate using a predetermined dose conversion coefficient.^28^ Since the external radiological impact of ^238^U series is predominantly attributed to radon’s progeny,^29^ RnDRs are essentially equivalent to the ^238^U dose rates within a few percent accuracy.

### 2.3 Sources of Contribution

**Figure 2:**
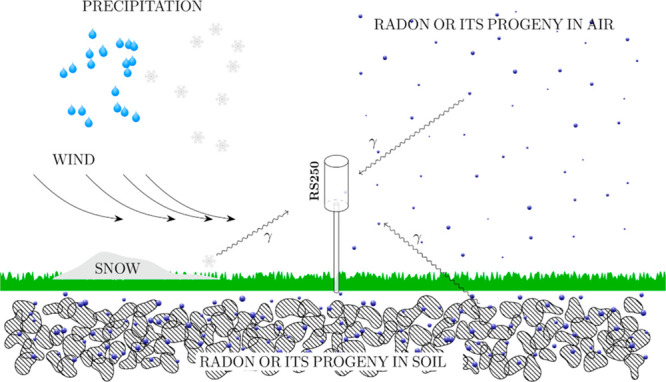
An illustration of the sources contributing to RS250 detection of 1.76 MeV gammas. The two main sources are radon and its short-lived progeny in the atmosphere and soil (pore space and grain). Precipitation can also be a temporary source of radon progeny. Wind- or temperature-gradient-driven air mass movement can also be a driving force to redistribute radon that originates from upper atmosphere or distant regions.

Owing to the nature of RnDR measurements, the 1.765 MeV γ rays may originate from various sources (Figure 2). These potential sources include ^214^B/^222^Rn present in the atmosphere, soil grains, and pore spaces, as well as precipitations. Moreover, radon transported from distant locations to the Arctic (as depicted by the wind in Figure 2) may also contribute.^30−33^

Given this study’s primary focus on seasonality and long-term trends, transient RnDR variations induced by precipitations (i.e., enhancement or depression) were initially eliminated through a data smoothing procedure. The enhancement results from the radon progeny that forms in the upper atmosphere, attaches to aerosol particulates, and then gets washed out to the ground by precipitation.^34^ It is followed by depressions lasting hours to days, depending on rainfall conditions, due to shielding from increased soil moisture.^1^

This study utilizes two aerosol radionuclide data sets for monitoring source locality. They are the ^210^Pb and ^212^Pb air concentration data collected from monitoring stations under the Comprehensive Nuclear-Test-Ban Treaty^32,33^ and Canadian Radiological Monitoring Network,^35^ respectively. The long half-life of ^210^Pb makes it valuable for tracing crossing-continent air mass transportation (over several thousand kilometers)^4^ into the Arctic. Meanwhile, ^212^Pb data are suitable for monitoring atmospheric phenomena at a local to subregional scale (distances of a few to several hundred km)^36^ because of the short half-lives of ^212^Pb and its parent radionuclide ^220^Rn.

Except for precipitation-induced and distant sources, other sources mentioned in Figure 2 are more likely to have local/regional origin. Based on mobility, they are divided into two categories. The first category includes stationary radon, found in soil grains or pore water. In the latter case, the diffusion distance is small as compared to source-to-detector geometry, making it effectively stationary. The second category is mobile radon, which can travel considerable distances naturally.

Stationary radon contributes to RnDR primarily from the top 20–30 cm soil layer and almost entirely from the top 50 cm layer (i.e., intermediate depth thereafter).^26,37^ Mobile radon, originating from as deep as a few meters below the ground surface, can reach the atmosphere.^1,38^ If it migrates to the intermediate soil layer or disperses into the atmosphere, it can also contribute to above-ground exposure. For radon gas originally produced within the intermediate layer, its upward movement within soil can increase the level of above-ground exposure. However, after reaching the atmosphere, it can either increase or decrease the overall exposure, depending on the combinational effects of changes in detection efficiency and attenuation strength.

### 2.4 Meteorological Influences

For local radon sources, if assuming undisturbed mineralogical and geological conditions, then the primary factors influencing RnDR are variations in meteorological conditions. The conditions of relevance are atmospheric temperature and pressure, soil moisture and temperature, and snow coverage on the ground.^1,38−41^

Mobile radon is susceptible to all of these conditions by means of affecting emanation, air–water partitioning, and vertical mobilizations in soil and the atmosphere. Consequently, radon concentrations and profiles in the soil pore space and the atmosphere vary over time. This results in a dynamic source-detector geometrical relationship with changing gamma detection efficiency and attenuation strength and thus varying outdoor RnDR. For stationary radon sources, water content in the soil, on the ground surface, and above ground are effective in attenuating γ rays, thereby affecting the RnDRs at detection sites. Collectively, these conditions are referred to as Soil and Above Water Content (SAWC).

For the above reasons, RnDRs in this study represent a complex environmental subject involving multiple sources and factors. Given limited meteorological data available and the intention of maximizing exploration of environmental effects, various meteorological data (Section 2.5) and three radiation monitoring data sets (Section 2.6) were used. The latter include ^40^K dose rates (KDRs) and the aforementioned ^210^Pb and ^212^Pb air concentration data.

### 2.5 Meteorological Data

One weather data set used in this study is the surface temperature analysis data from Goddard Institute for Space Studies.^42^ It contains a century worth of data on temporal temperature changes or anomalies in reference to the 1951–1980 temperature mean. Local weather data were collected by Environment and Climate Change Canada (ECCC)^43^ stations close to the two FPS sites. These include hourly temperature and pressure data, daily precipitation records, and snow depth records collected at the Yellowknife (Resolute) station between 2005 (2013) and 2022. Further details are available in the Supporting Information.

### 2.6 Supporting Radiation Monitoring Data

In this study, soil temperature and moisture data, as well as soil/air radon concentrations, are unavailable. To make the most of FPS data for climate change research, other supporting data were utilized to infer variations and impacts of the missing environmental variables.

The ECCC atmospheric temperature serves as an indicator of seasonal soil temperature variation. These two temperatures are typically correlated on a long-term scale,^44^ albeit with a time lag. Furthermore, soil temperature data from locations within a 70 km radius of Yellowknife^20,21^ were available to infer likely soil thaw-depth and temperatures at depths of 50 and 100 cm near Yellowknife station.

Soil moisture variations can be inferred from snow depth, melting conditions, rainfall history, and ^40^K exposure. The latter is a popular radiation-based technique to sense water content changes in media.^45,46^ In FPS, the ^40^K dose rate is due to the 1.460 MeV gammas almost exclusively from soil potassium, hence varying primarily with SAWC conditions. Furthermore, because this energy is close to the 1.765 MeV of ^214^Bi, their transmission strengths differ within 4.1% in water of 10 to 100 mm in thickness (equivalent to 10–100 cm of snow, assuming a snow density of 10%). This range covers the highest recorded snow depth of 80 cm in Yellowknife since 1955.^47^ Hence, varying KDR can be used to reflect the SAWC impacts experienced by stationary radon within a few percent accuracy.

Gaseous radon and thoron (^220^Rn from thorium decay series) in soil exhibit similar exhalation responses to environmental factors.^48,49^ For instance, soil temperature positively affects their exhalation rates.^38,50−52^ Additionally, their exhalation rates increase with rising soil water content up to a certain level, beyond which they gradually decrease with further increases in soil moisture.^51−53^ Therefore, aerosol data of thoron’s progeny ^212^Pb can be used to infer radon gas concentration variations in soil/atmosphere. Moreover, studies have shown that soil water content is the primary factor affecting their concentrations.^48,51^ So the ^212^Pb data also reflect changes in SAWC conditions within the topsoil layer of a few centimeters, where thoron primarily originates.

### 2.7 Analysis Methodology

RnDR long-term trends were studied across three time scales: yearly, quarterly, and on summer dry days (see Supporting Information Table S3 for all trend and correlation results). Yearly analysis relies on annual averages without discerning effects of various factors, whereas quarterly analysis examines data from the same quarter over a number of years. Lastly, the summer-dry-days analyses specifically target days with above-freezing temperatures and no precipitation.

Simple linear regression analyses were conducted for long-term trend estimates, including the slope of fit, its standard error, and p-value (*p*), as well as coefficient of determination (r^2^). The correlations between RnDR and variables were assessed through Pearson correlation coefficient (r) in univariate linear regression. The adjusted R-squared (adj_R^2^) in multivariate regression analyses was determined to quantify the relative importance of variables/features, and the fitted slopes (i.e., coefficients βs) at the maximum adj_R^2^ were used to quantify relationships. The Supporting Information provides additional details.

## 3 Results and Discussion

### 3.1 Decomposed ^222^Rn Dose Rate

To study RnDR’s seasonality and long-term patterns, it is necessary to eliminate the short-term fluctuations caused by precipitations. This was achieved using a low-pass Butterworth filter,^54^ which offers a maximally flat response for smoothing long-term variation and handles data edges through a forward–backward process. The original RnDRs were then decomposed into two components: smoothed trend and short-term precipitation (and background) components. The trend components expectedly exhibit a smooth pattern and clear seasonality and are further used in subsequent analyses. Additional details are available in the Supporting Information.

**Figure 3:**
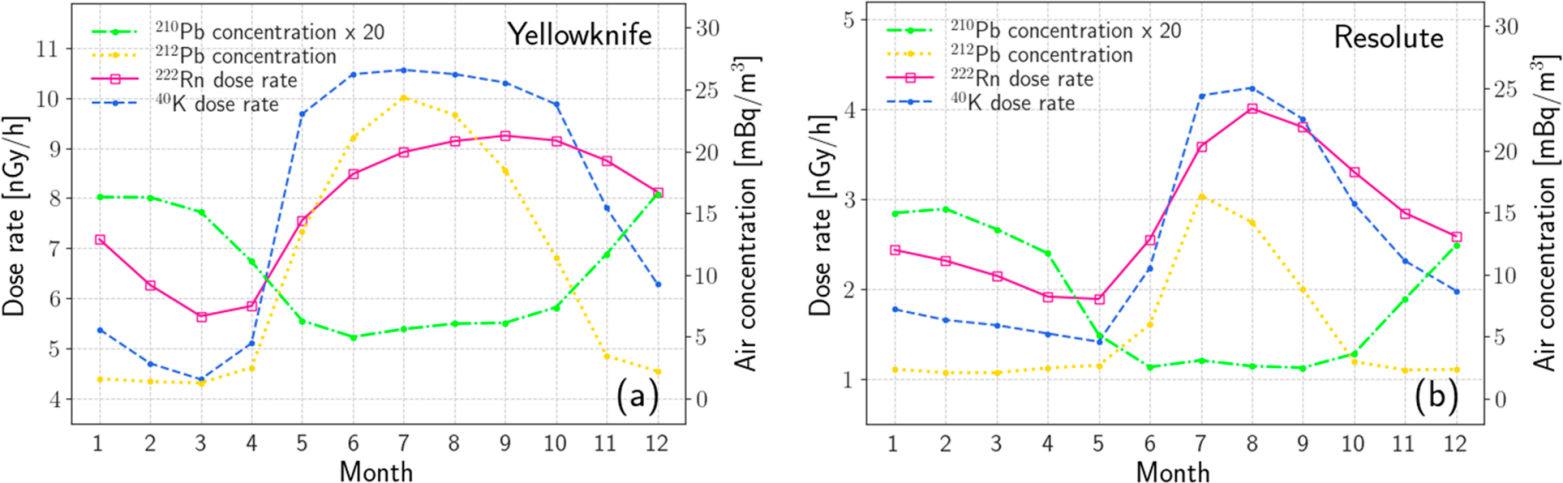
Monthly means of RnDR, ^40^K dose rate, and air concentrations of ^212^Pb and ^210^Pb. To accommodate the ^210^Pb data in these plots, its concentration was scaled up by a factor of 20.

In terms of radiological risk to human health, the smoothed RnDRs have monthly averages ranging from about 5.7 to 9.2 nGy/h in Yellowknife and from about 2.0 to 4.0 nGy/h in Resolute (Figure 3). The different levels can be partially attributed to the mineralogical characteristics (i.e., typical rock types and levels of uranium concentration in them) of each area. Yellowknife, located in the NWT of Canada, is known for rich uranium deposits and highly uranium-bearing rocks.^55,56^ Differently, Resolute’s ground surface is predominated by sedimentary rocks such as limestone and dolomite,^57,58^ which typically do not have much uranium enrichment.^59^ Being a sidenote, the indoor radon concentration in Yellowknife is among the highest in the Canadian Arctic.^60^

### 3.2 RnDR Seasonality

Principal component analysis was conducted as an initial inspection on the variability of available weather conditions (see Supporting Information for details). Results suggest that temperature and snow depth are key variables driving overall environmental variability. They both also closely correlate with smoothed RnDR, making them the most relevant for subsequent analyses.

Figure 3 shows RnDR seasonality in both regions (more discussions in the Supporting Information), which covers a study period from 2005 to 2022 for Yellowknife and from 2013 to 2022 for Resolute.

Generally speaking, RnDR and KDR upturn their trends in springtime as snow melts and water loss occurs above ground. Following are rapid rises over 1–2 months, primarily caused by quick drying conditions of SAWC. Interestingly, RnDR did not keep pace with KDR increases. The discrepancy is attributed to radon gas entering the atmosphere and becoming significantly diluted, contributing less to RnDR compared to that otherwise trapped in soil. The ^212^Pb concentration trace confirms increased noble gas emissions from soil during this period.

At both stations, KDR peaks in the summer, lasting for 1–2 months before entering a suppression phase. This signals the time when the soil has thawed beyond the intermediate depth. In Yellowknife, suppression periods for KDR and ^212^Pb concentration coincide due to common underlying causes of increased soil moisture during rainy seasons and winter snow accumulation. Resolute’s KDR, however, pivots downward later than ^212^Pb concentration. In Resolute’s cold and dry summer, a thin layer of frozen ground surface is effective in suppressing soil gas release but has minimal impact on attenuating gammas. Later as snow accumulates on the ground, KDR starts a noticeable fall.

Decreasing ^212^Pb concentration in the atmosphere implies an increasing soil radon gas concentration, which modulates the overall RnDR. As a result, after KDR falls, Yellowknife’s RnDR continues to rise for months, whereas Resolute’s RnDR decreases more gradually. As soil freezing deepens, soil radon behaves more like ^40^K in mobility, and the RnK (RnDR to KDR) ratio gradually summits and stabilizes. Yellowknife’s RnK ratios are 1.28 in December, 1.34 in the next two months, and 1.29 in March. In Resolute, RnK ratios rise steadily from 0.98 in September to 1.33 in December before plateauing at ∼1.40 from January to March. In these instances, maximal ratios mark periods of year when refrozen soil exceeded intermediate depth. Springtime drops in RnK ratios signal soil gas release revival.

The long-distance transportation process (see the ^210^Pb traces) is active during winter months and dormant in warm seasons, consistent with earlier findings.^32,33^

### 3.3 Year-Based RnDR Long-Term Trends

**Figure 4:**
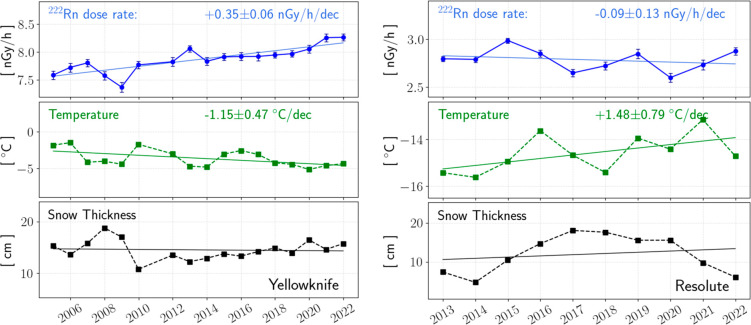
Annually averaged RnDRs, temperatures, and snow depths at the Yellowknife and Resolute stations. The lines are linear regression trends, and the legends shown at the top and middle panels are the fitted slopes. The error bars shown in dose rate plots are the standard errors of annual mean. The 2011 data was removed in Yellowknife plots due to incomplete dose rate measurements that year. At both stations, regression lines shown in snow depth plots are solely visual guides due to high variability in data.

Yellowknife’s RnDRs (Figure 4-left) exhibited a discernible upward trend from 2005 to 2022. Regression analysis indicates an increase rate of +0.35 ± 0.06 nGy/h per decade (nGy/h/dec). Meanwhile, its temperature decreased by −1.15 ± 0.47 °C per decade (°C/dec), contradicting the forty-year trend of +0.33 °C/dec (see the Supporting Information). Regarding snow thickness, high variability during the initial several years results in a poor linear fit with no trend.

In Resolute (Figure 4-right), RnDR showed a slight decline from 2013 to 2022 with little statistical significance (r^2^ = 0.06). Meanwhile, Resolute experienced warming at a rate of +1.48 ± 0.79 °C/dec, more than doubling the rate of +0.66 °C/dec observed from 1980 to 2020 (Supporting Information). No trend was found on snow depth.

RnDR was weakly anticorrelated (i.e., inversely correlated) with temperature in Yellowknife (r = −0.37), whereas no correlation was found in Resolute (r = −0.04). Meanwhile, snow thickness was moderately anticorrelated with RnDR at both stations.

The annual means aggregate various fluctuations and meteorological impacts into a single yearly value, hindering the differentiation and study of specific environmental factors or time scales. To better understand trends and correlations, data were divided into four quarters: Q1 (January to March), Q2 (April to June), Q3 (July to September), and Q4 (October to December).

### 3.4 Yellowknife Quarterly Based RnDR Long-Term Trends

**Figure 5:**
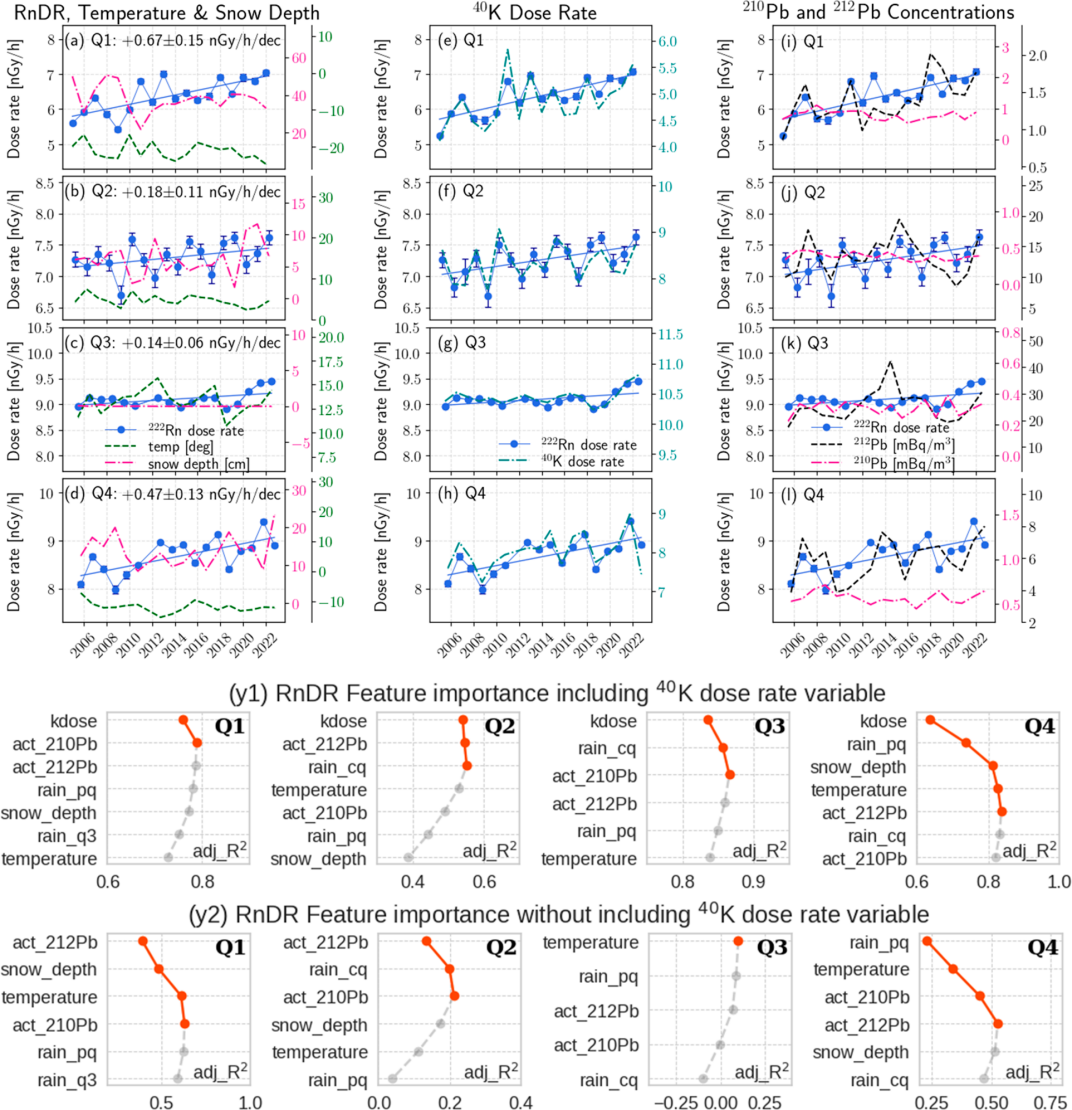
Plots (a-l): quarterly averaged data long-term trends in Yellowknife. Blue lines are the fitted slopes on RnDRs, and decadal trends are given in the legends of plots a-d. The RnDR data and blue lines in other plots are only for comparison purposes. The error bars in the RnDR data are the standard errors of the quarterly mean. The 2011 data was removed in all Q3 and Q4 plots due to incomplete measurements in these two quarters. Plots (y1-y2): feature relevance in the scenarios of including (y1) and excluding (y2) the ^40^K dose rate in modeling RnDR data. Variables on the *y*-axis are arranged in a descending order in relevance starting with the most significant one at the top. The *x*-axis represents the overall adj_R^2^ updated each time as a variable is added to the model, whereas the change in adj_R^2^ is the unique variance that this variable accounts for beyond previously included ones. Features that positively contribute to modeling (i.e., change in adj_R^2^ > 0) are displayed as red dots and otherwise as gray dots. Variables rain_cq, rain_pq, and rain_q3 refer to hourly rainfall in the current quarter, previous quarter, and third quarter, respectively. The variable kdose represents the ^40^K dose rate. The prefix “act_” refers to air concentration.

As shown in Figure 5a-d and Table S3, RnDR trended upward in all quarters except Q3 (non-Q3). In these quarters, temperature fell by −1.0 to −1.5 °C/dec, and snow depth exhibited flat trends due to large year-to-year fluctuations. Two winter quarters (Q1 and Q4) saw the largest RnDR increases, thereby primarily accounting for the annual rise (Figure 4). Local radon emission also increased in these two quarters, as indicated by the ^212^Pb data (Figure 5i-l and Table S3). In Q3, the RnDR regression results were invalid due to highly skewed data over the past few years.

In non-Q3 quarters, the RnDR was inversely correlated with snow depth and ^210^Pb concentration. RnDR correlated negatively with temperature in two winter quarters but positively in other quarters. RnDR was strongly correlated with both KDR and ^212^Pb concentrations in almost every quarter, indicating these variables were concurrently influenced by common fluctuating SAWC and thus closely followed each other.

Notably, the two winter quarters observed the most rapid increases in both KDR and RnDR, which was not explained by snow depth variation, which did not decline. Therefore, other forms of SAWC must have declined over the years in these quarters. Additionally, the rising ^212^Pb concentration amid declining temperatures also favors the hypothesis of reduced SAWC in soil, at least in the top layer.

Figure 5y1-y2 shows two scenarios of feature relevance analyses, one with and one without KDR variable in modeling data. The *y*-axis shows variables arranged in descending order of relevance, with the most significant one at the top. The *x*-axis represents the overall adj_R^2^ updated each time a variable is added to the model, whereas the *change* in adj_R^2^ is the unique variance that this variable accounts for. All regression results, including adj_R^2^ contributions and RnDR-variable relationships (βs), can be found in the Supporting Information.

In Figure 5-y1, the optimal model explains about 79%, 55%, 87%, and 84% of RnDR variance in Q1 to Q4, respectively. In each case, KDR is the primary contributor, highlighting the dominance of stationary radon. Beyond that, other relevant variables then explain the variation in mobile portion of RnDR (mRnDR). These mainly involve two types of variables: mobile sources represented by ^210^Pb and ^212^Pb air concentrations and precipitation related variables such as rainfall and snow depth.

In Q1, when local emission is at the lowest, the distant source (represented by ^210^Pb concentration) was identified relevant with a negative β. In snow-melting quarter Q2, local radon emission (represented by ^212^Pb air concentration) became relevant to mRnDR. The rainfall impact (rain_cq) in Q3 was relevant with a positive β, suggesting radon trapping effect overweighs gamma attenuation impact. In Q4, in addition to snow depth, prewinter precipitation (i.e., rain_pq) was also relevant (negative β) as entering the soil and sustaining throughout winter months. Given that snow and frozen ground surface in Q4 already effectively confine radon in the soil, prewinter water content in the soil thus primarily serves as a gamma attenuating medium. Negative βs of ^212^Pb concentrations in winter quarters suggest increased mRnDR as soil gas release is suppressed.

Figure 5-y2 shows feature relevance without considering KDR, thereby referencing the total RnDR from both stationary and mobile radon. When compared to Figure 5-y1, all quarters showed a general decrease in adj_R^2^, with winter quarters being relatively better explained due to low mobility of soil radon gas. Low adj_R^2^s suggest the existence of additional factors beyond those already considered in explaining the RnDR long-term variations.

Without KDR, ^212^Pb concentration has the highest relevance in Q1 and Q2. This is because in these two quarters SAWC conditions vary significantly in the shallow soil layer and above, the range that ^212^Pb concentration can effectively monitor. Positive β coefficients were found in both cases. In Q3, temperature was determined to be the only relevant variable, with a β of +0.047 ± 0.028 (nGy/h)/°C, the only positive coefficients of all quarters. In Q4, rainfall from the previous quarter and temperature are the most relevant with negative βs. With a negative coefficient, rainfall from the previous quarter demonstrated its impact as a gamma attenuating medium throughout winter months.

### 3.5 Resolute Quarterly Based RnDR Long-Term Trends

**Figure 6:**
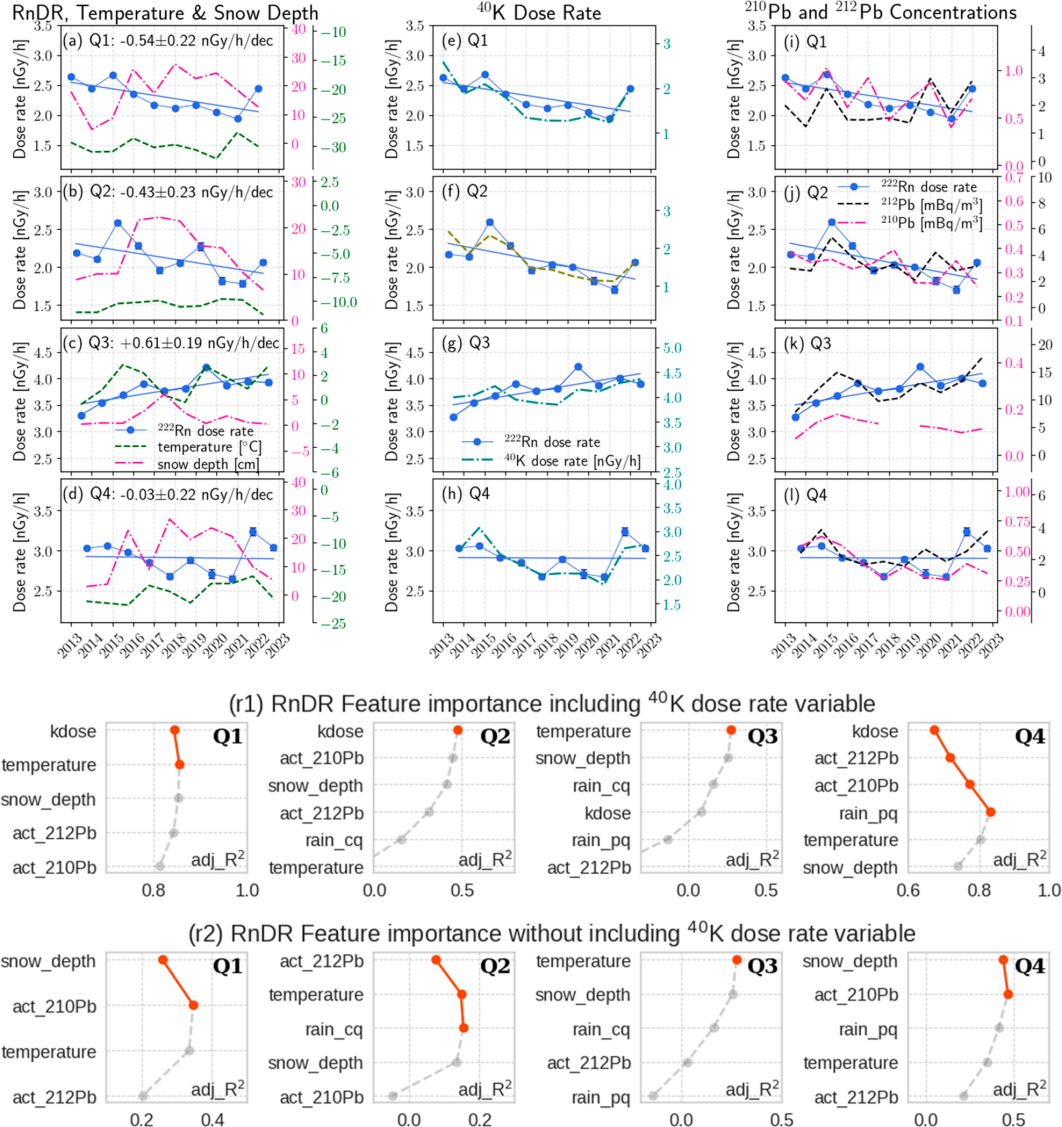
Plots (a-l): quarterly averaged data and long-term trends in Resolute. The blue lines are the fitted slopes on RnDRs, and the decadal trends are given in the legends of plots a-d. The RnDR data and blue lines in other plots are only for comparison purposes. The error bars in the RnDR data are the standard errors of quarterly mean. The 2018 ^210^Pb data point is missing in plot k due to incomplete data in this quarter. Plots (r1-r2): feature relevance in the scenarios of including (r1) and excluding (r2) the ^40^K dose rate in modeling RnDR data. Variables on the *y*-axis are arranged in a descending order of relevance starting with the most significant one at the top. The *x*-axis represents the overall adj_R^2^ updated each time as a variable is added to the model, whereas the change in adj_R^2^ is the unique variance that this variable accounts for beyond previously included ones. Features that positively contribute to modeling (i.e., change in adj_R^2^ > 0) are displayed as red dots and otherwise as gray dots. Variables rain_cq and rain_pq refer to hourly rainfall in the current and previous quarters, respectively. The variable kdose refers to the ^40^K dose rate. The prefix “act_” refers to the air concentration.

Resolute’s annual mean temperature rise (Figure 4) was found to be primarily caused by increases in Q3 and Q4 (see Table S3 and Figure 6a-d). In Q3, RnDR increased by +0.61 ± 0.19 (nGy/h)/dec, with the only positive temperature-RnDR correlation in a year. In other quarters, RnDR’s trend analyses are susceptible to a few influential data points, such as the rebounds in 2021 and 2022, which are linked to reduced snowfall during Q4 of 2021 (see the Supporting Information for more details).

Such interquarterly meteorology-RnDR correlations are not uncommon in Resolute. Due to a short and dry summer, snowfall is the SAWC form impacting RnDR the most. Furthermore, snowfall in Q4 impacts RnDRs during winter and spring significantly and long-lastingly, since the main snow season in Resolute runs from September to November. Snow depth was moderately anticorrelated with RnDRs in Q1 and Q4.

Both KDR and ^212^Pb concentrations are positively correlated with RnDR in all quarters. For ^210^Pb data, a clear downward trend was found in non-Q3 quarters (the Q3 trend was not assessed due to incomplete data in 2008).

KDR was found to be most relevant (Figure 6-r1) in modeling RnDRs of non-Q3 quarters. In Q3, temperature was determined to be the only relevant feature with a β coefficient of +0.115 ± 0.055 nGy/h per °C. In fact, this quarter’s KDR had a slightly weaker correlation with RnDR than temperature (see Table S3). Additionally, the Q3 regression results in Figure 6r1-r2 are identical, because KDR was considered as insignificant in both cases.

To mRnDR (Figure 6-r1), the temperature in Q1 was only marginally relevant. No features besides KDR were relevant in Q2. In Q4, rainfall from quarter Q3 (denoted by rain_pq) significantly impacted mRnDR with a negative β. Similar to the Yellowknife case, Q3 rainfall affects the mRnDR in Q4 primarily through gamma attenuation rather than radon gas confinement. Meantime, ^212^Pb concentration in Q4 was also relevant with a negative β, indicating that radon gas tends to enhance mRnDR when confined in the soil as opposed to dispersing into the atmosphere. Moreover, the ^210^Pb concentration was found to contribute approximately 6% adj_R^2^ to mRnDR variation. Its negative β can be an indication of the dilution effect rather than a supply if a distant source contains less radon content than local emissions. Further study is required to gain a better understanding.

Without incorporating KDR into the analyses (Figure 6-r2), Q4 results illustrate that both the snow depth and ^210^Pb concentration were relevant to the overall RnDR, together accounting for about 46.6% RnDR variation. Snow depth has a negative β. Regarding the ^210^Pb concentration, it was positively associated with the overall RnDR from both stationary and mobile radon, albeit not statistically significant. While this association is anticipated to be mainly accidental rather than causal, exact reasons are being investigated. Similar results were obtained in Q1, another cold quarter in Resolute. In Q2, the available variables were far less adequate in explaining RnDR variations (maximum adj_R^2^ of ∼15%). The ^212^Pb air concentration was relevant here, because it is an effective indicator of the SAWC variation in springtime.

### 3.6 Long-Term Trends on Warm Dry Days

According to Figures 5-y2 and 6-r2, the temperature was the only relevant variable in Q3 at both stations. A simplified scenario excluding snow coverage and precipitation was studied to determine if fewer environmental factors could lead to a better constrained temperature-RnDR relationship. However, the results showed no improvement (see the Supporting Information for details).

### 3.7 Climate Change Implications and Radiological Risks

Amid global climate change, temperatures in Canada’s northern region trended unevenly over time and across locations. Yellowknife’s temperature has declined over the past two decades by −1.15 ± 0.47 °C/dec, diverging from its long-term trend of +0.33 °C/dec Differently, Resolute has been warming by +1.48 ± 0.79 °C/dec over the past decade, more than doubling the +0.66 °C/dec rate during 1980–2020. The rapidest changes occurred in Yellowknife’s non-Q3 quarters and Resolute’s Q3-Q4.

The climate change impacts on the RnDR were observed in both regions. In Q3, both Yellowknife (Figure 5-y2) and Resolute (Figure 6-r2) showed a positive temperature-RnDR relationship. Notably, Resolute’s Q3 had a stronger β (+0.115 nGy/h/°C), indicating a faster RnDR increase compared to Yellowknife for each degree of temperature rise over years. Considering Resolute’s relatively low uranium concentrations, mineralogical factors were ruled out in explaining this disparity.

In Resolute, since all days above freezing fall in Q3, soil thawing in this quarter primarily occurs within the RnDR-effective layer, extending from the topmost layer down to its maximum active layer depth. This sensitivity enables RnDR to dynamically respond to prolonged thawing or/and a deepened active layer likely caused by warming Q3. Differently, Yellowknife experienced soil thawing beyond the intermediate depth during most Q3 days, resulting in slower RnDR growth or a plateau phase (Figure 3-a). Furthermore, Resolute’s temperature-RnDR relationship was reinforced by its dry summer, coarse terrain, and nearly bare ground coverage. These conditions allow the shallow soil layer to respond closely and directly to atmospheric temperature changes, with little interference from soil moisture or vegetation. Conversely, Yellowknife’s muskeg-style terrain and wetter summer hampered soil’s temperature response, weakening the observed effect. These divergent scenarios demonstrated the influences of climatic characteristics and geological features, along with temperature, in research on climate change impacts on RnDR and other soil-related topics.

In other quarters, RnDR was more impacted by SAWC conditions, and their relevance varied with seasonal conditions (e.g., the temperature-driven soil thaw-freezing cycle and snowmelt) and precipitation patterns in the region. For example, in Q4, Yellowknife’s RnDR was heavily influenced by prewinter rainfall, whereas in Resolute it was snow depth. Yellowknife’s abundant warm-season rainfall, especially before winter freezing, can be stored in soil, causing lasting effects on RnDR. Differently, in Resolute, dry summer and bare ground make snow depth the key factor in winter quarters. These contrasting scenarios highlight how changes in precipitation amount and pattern, whether naturally occurring or caused by climate change, impact RnDR and its long-term behavior.

Based on these results, the outdoor radon exposure and climatic impacts were evaluated in terms of the effective dose. Yellowknife’s RnDRs averaged annually 7.86 ± 1.34 nGy/h from 2005 to 2022, while in Resolute, it was 2.78 ± 0.93 nGy/h during 2013–2022. After applying a conversion coefficient of 0.7 Sv/Gy,^1^ the yearly effective dose totaled 48.19 ± 8.22 uSv in Yellowknife and 17.05 ± 5.70 uSv in Resolute. Over the last two decades, Yellowknife’s exposure increased by 2.15 ± 0.37 uSv/dec (or ∼4.5%/dec), with Q1 the fastest at ∼11%/dec. In Resolute, the fastest increase occurred in Q3, at ∼16%/dec. These levels and increases are comparable to the typical outdoor-radon inhalation exposure in Canada but significantly lower than indoor inhalation exposure.^61^ So, from an external exposure perspective, they pose little practical health risks to the public.

In addition to outdoor-radon external exposure (i.e., RnDR), the ^212^Pb results provide insights into variations and long-term trends in soil gas emissions. Soil gas emissions rise during springtime and decrease in the winter. Frozen topsoil, increased moisture, and precipitation are effective ways to reduce soil gas emissions. Over the years, Resolute’s Q3 and Yellowknife’s two winter quarters have experienced increased soil gas emissions, including radon gases. The former is likely due to a deepening active layer, whereas the latter is associated with changing SAWC conditions. From a health perspective, elevated soil radon emissions indicate increased outdoor-radon inhalation exposure.

Furthermore, combined supporting data sets helped evaluate changes in soil moisture and porosity, crucial factors influencing radon gas migration from soil into indoor environments.^62,63^ While the ^212^Pb concentration and KDR variations sense SAWC changes down to different soil depths, snow depth confirms whether changes stem from the ground or within the soil. In Yellowknife’s winter quarters, rising ^212^Pb and KDR trends, along with nontrending snow depth, imply reduced water content in soil over time. This may have led to increased indoor radon concentration due to enhanced soil permeability, requiring direct measurements to confirm this, though. Lastly, ^210^Pb results suggest a weakening long-distance transportation process over years during winter quarters in both regions.

This research studied Arctic’s outdoor radon exposure amid climate change. It enhanced our understanding of dynamic radon exposure scenarios and complex environmental factors and processes at play. Climatic impacts were empirically confirmed and quantified. Various data sets helped facilitate the environmental investigation to the fullest extent possible. This approach proved highly effective and holds scientific significance in studying climate change impacts related to soil gas migration and release. More measurements and research are preferred for a better understanding.

## Supporting Information Available

Meteorological data; Analysis methodology; Data decomposition; Principle component analysis; Annual radon dose rate cycle in Yellowknife; Annual radon dose rate cycle in Resolute; Interquarterly correlation in Resolute’s RnDR; Long-term trends on warm dry days; Trend and correlation results; Feature relevance with multivariate linear regression analyses; Other possible influencing factors (PDF)
