# Supplementary material for: Outdoor Radon Dose Rate in Canada’s Arctic amid Climate Change
Source: Environ Sci Technol. 2024 Jun 22;58(26):11309–19. doi: 10.1021/acs.est.4c02723 (PMC11223471; doi:10.1021/acs.est.4c02723)
Supplement: Supplementary file 1 — es4c02723_si_001.pdf [file es4c02723_si_001.pdf]

Supporting information for

Outdoor Radon Dose Rate in Canada's Arctic amid Climate Change

Chuanlei Liu[a], Jing Chen[a], Weihua Zhang[a] and Kurt Ungar[a]*

[a]*Radiation Protection Bureau of Health Canada, 775 Brookfield Rd, Ottawa, ON K1A 1C1, Canada*

*Email: Chuanlei.Liu@hc-sc.gc.ca

The supporting information file contains

Number of pages: 24

Number of figures: 9

Number of tables: 5

Table of Contents

SI-1: Meteorological Data S3

SI-2: Analysis Methodology S5

SI-3: Data decomposition S6

SI-4: Principle Component Analysis S8

SI-5: Annual Radon Dose Rate Cycle In Yellowknife S10

SI-6: Annual Radon Dose Rate Cycle In Resolute S16

SI-7: Inter-quarterly correlation in Resolute's RnDR S20

SI-8: Long-term Trends on Warm Dry Days S21

SI-9: Trend and correlation results S24

SI-10: Feature Relevance With Multivariate Linear Regression Analyses S24

SI-11: Other possible influencing factors S26

SI-1: Meteorological Data

The Surface Temperature Analysis (GISTEMP v4) data, obtained from the Goddard Institute for Space Studies, contain century's worth of data on the temporal temperature changes or anomalies in reference to the 1951-1980 temperature mean. These data aid in a better understanding of the long-term temperature trend within the two Arctic regions as compared to the changes observed during the relatively brief period of FPS (Fixed-Point Surveillance network) measurements.

As shown in Figure S1, GISTEMP data indicate that the last decade has been the hottest ever observed for all three zonal regions, with rapid temperature increases starting in about 1980. The year of 2020 saw the highest average temperature for the northern hemisphere ever recorded (+1.36 °C above the reference average), while the Arctic experienced its warmest year since 1880 (+3.24 °C above average) in 2016. According to a linear fit on the 1980-2021 data, the Arctic was warming at a rate of +0.686 °C/decade, more than doubling the rates of +0.308 °C/decade found in the mid-latitudes and +0.282 °C/decade for the entire northern hemisphere.

The local weather data in Yellowknife and Resolute were collected by the Environment and Climate Change Canada (ECCC) stations close to the two FPS sites. These include hourly temperature and pressure data, daily precipitation records and snow depth records. Based on the temperature data collected since 1953, Yellowknife and Resolute have mean temperatures of -4.543 °C and -15.681 °C, respectively. Additionally, significant year-to-year fluctuations is clear in both areas, as shown by the ECCC data in Figure S1. Despite this, the long-term trends since 1980 were consistent with the warming rate in each respective latitude zone. Over the latest forty years, Resolute has warmed up at a rate of +0.66 °C/decade while Yellowknife has seen temperature rise as much as +0.33 °C/decade.

The study periods for this work run from 2005 to 2022 in Yellowknife and from 2013 to 2022 in Resolute. To associate FPS' outdoor Radon Dose Rates (RnDRs) with local weather conditions, the ECCC data collected during the same periods were extracted. These comprise records of temperature, precipitation, snow depth on the ground and atmospheric pressure. The bottom two

plots in Figure S1 show the temperature and precipitation conditions over the years of FPS measurements. In contrast to the forty-year trends, temperature data here, despite of the short-term variability, suggest that Yellowknife has actually been cooling since 2005, while Resolute has warmed in the last ten years twice as fast as the rate seen since 1980.

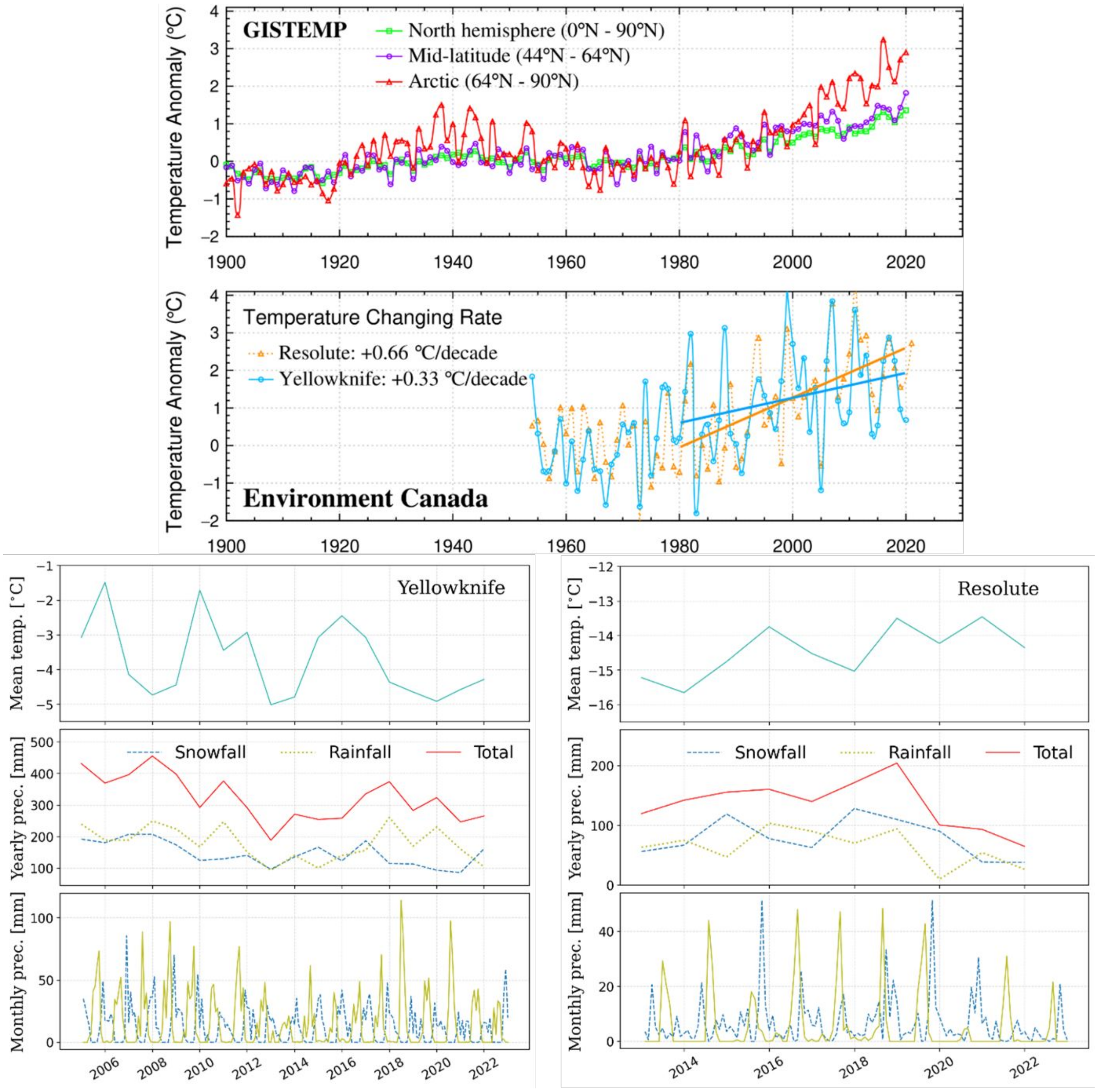

Figure S1: Top plots: the GISTEMP data showing the annual temperature anomalies for north hemisphere, and its mid-latitude and arctic regions, while the ECCC data show the annual temperature anomalies for Yellowknife and Resolute. Bottom plots: the annual-mean temperature, and annual-sum and monthly-sum precipitation records in

Yellowknife (left) and Resolute (right). Here the snowfall amount was converted to water by assuming a snow water equivalent of 10%.

The precipitation in Yellowknife is moderate. Based on past 70-year ECCC data, Yellowknife received about 307 mm of precipitation annually, of which about 163 mm came in the form of rainfall. Here the amount of snowfall was converted to mm from cm by assuming a snow water equivalent of 10%. Resolute has a polar desert climate, with an average annual precipitation of 152 mm. Rainfall usually occurs from June to August during the summer, whereas snowfall can take place any month of the year. The annual rain precipitation was averaged to 68 mm based on ECCC data from 2013 to 2021, an increase of 10 mm with respect to the average over seventy years of data.

SI-2: Analysis Methodology

Simple linear regression analysis was conducted to estimate the long-term trend, including slope of fit, standard error and p-value (p), as well as coefficient of determination (r^2). Coefficient r^2 represents the proportion of total variance that can be explained by the model. The standard error measures slope accuracy, whereas p-value indicates the probability of the estimate being compatible with null hypothesis (i.e., zero slope). In this study, the 68% (1-sigma) and 95% (2-sigma) rules were employed on p-value to indicate the statistical significance of a trend. A trend is highly confident/significant if its p-value is below 0.05, while low confidence when a p-value is between 0.05 and 0.32. Any trends with a p-value greater than 0.32 are deemed insignificant.

The correlations between RnDR and environmental factors were studied using both univariate and multivariate linear regression analyses. In the univariate analysis, the Pearson correlation coefficient (r) was determined to describe associations between RnDR and each factor. A strong correlation is deemed if $r > 0.7$, while a poor/weak correlation occurs when $r < 0.3$. Moderate correlations fall between these two thresholds.

Multivariate regression analyses evaluate the relative importance (or relevance) of multiple variables (or features) and simultaneously quantify their associations with RnDR. In this work,

relevance was determined using the adjusted R-squared (adj_R^2) and ranked using a forward selection technique. The selection started with a linear model including only one feature at a time and looped through all features to identify the one with highest adj_R^2 as the most relevant. Then, selection considered all possible bi-variate models by adding each of the remaining variables into the model. The variable with the largest adj_R^2 when added into the model was then considered the second most relevant feature. This process continued by adding more variables until every variable was considered. The fitted slopes (i.e., coefficients βs) obtained at the maximum adj_R^2 quantify the relationship between RnDR and variables. Note that unlike the normal R^2, the adjusted R^2 considers the number of variables and penalizes the inclusion of irrelevant ones. It may decrease or become negative if an irrelevant variable is added, especially when the sample size is small compared to the number of variables [Tabachnick and Fidell, 2007].

In feature relevance analyses, two scenarios were considered, one with and the other without KDR variable included in the regression model. In the former case, since KDR can approximate the stationary portion of RnDR variation, the identified features beyond it (i.e., all variables except for KDR) then stands out the variables most relevant to the non-stationary radon sources such as mobile radon or long-distance source. On the contrary, the latter case is a better option for identifying and assessing features that affect both mobile and stationary radon.

SI-3: Data decomposition

To mitigate precipitation-induced effects in RnDR, a few data filtering algorithms have been tested in this study, including the moving average method, the Savitzky-Golay (SG) filter [Savitzky and Golay, 1964; Press and Teukolsky, 1990], and a low-pass Butterworth filter. The moving average method is effective and straightforward, but it suffers phase shifting and feature attenuation issues. The SG filter utilizes local least squares polynomial approximation to better capture the shape of precipitation feature, such amplitude and duration. However, it is susceptible to artifacts at data boundaries. The low-pass Butterworth filter, implemented using the sosfiltfilt function in python (https://docs.scipy.org/doc/scipy/reference/signal.html), was

ultimately used in this study. This is because this method provides a maximally flat response for smoothing the long-term variation, and its forward-backward filtering process outperforms the other two algorithms at handling data edges. The filter was fine-tuned to preserve the smoothness of the rainy seasons while retaining the features of transitional periods. The process eventually ends up with two components: smoothed trend and short term precipitation (and background) components.

Figure S2 displays the times series of the hourly-averaged RnDRs in Yellowknife (left) and Resolute (right), as well as the decomposed components obtained using the Butterworth method. The red lines shown in the top and middle panels represent the trends after smoothing out the precipitation contributions as seen in the bottom panels. The spikes observed in the precipitation components are attributed to rainfall or snowfall events, which can cause several-fold increase in the RnDRs under certain circumstances. In contrast, the trend components expectedly exhibit a smooth pattern and show clear seasonal variations.

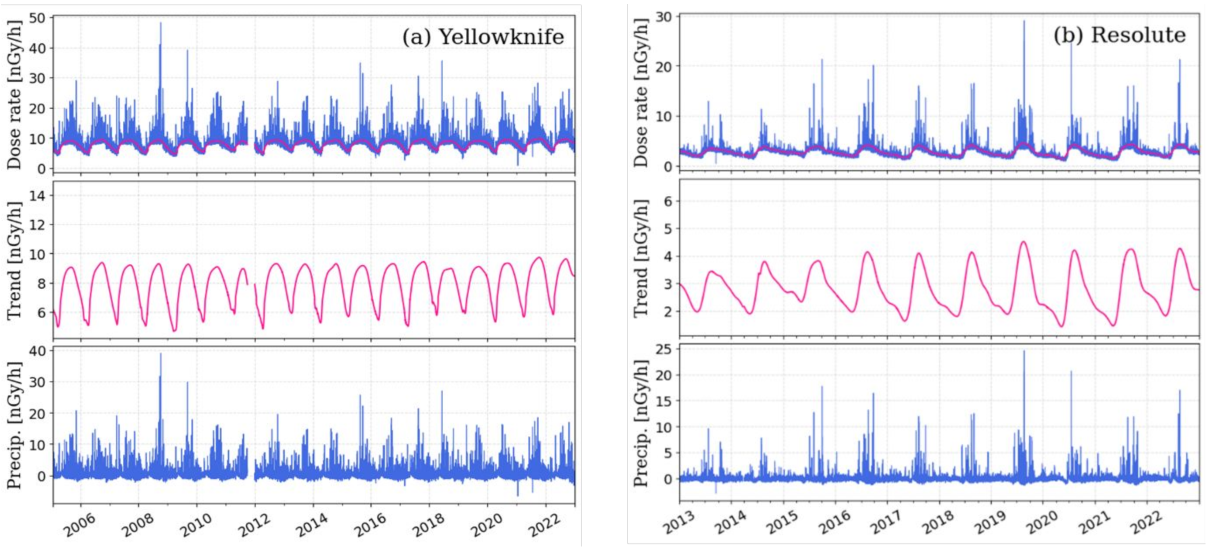

Figure S2: hourly-averaged RnDRs (original) and their respective decomposed components in Yellowknife (a) and Resolute (b).

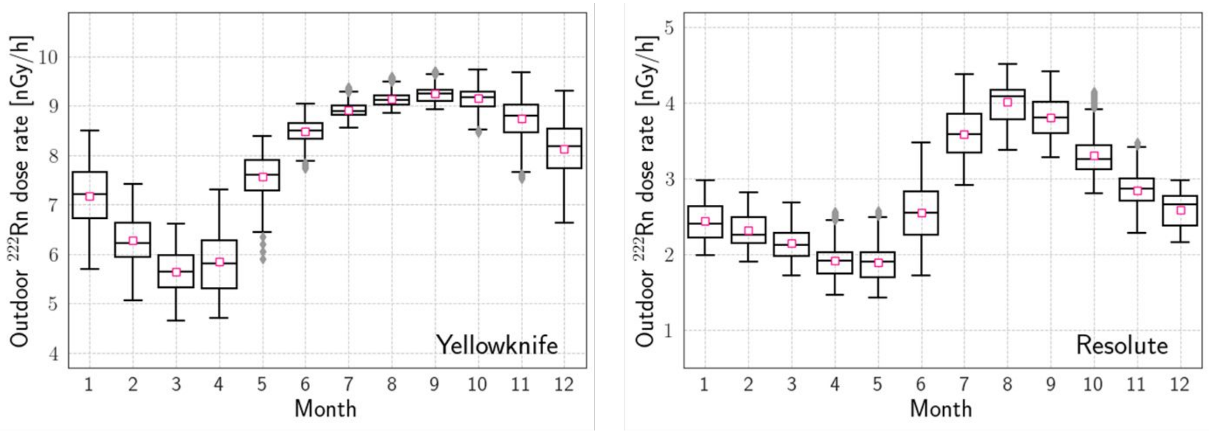

Figure S1: Monthly averaged radon dose rates over the measurement periods. The boxes depict the range of the central 50% of dose rates (i.e., between 25th and 75th percentile) in each month. The central line (square) within the box marks the median (mean) value. The whisker either captures the data extreme or marks the 1.5-time IQR position away from each side of box limit. Data beyond whisker are treated as outliers and shown as gray dots

With these smoothed components, the two whisker plots (Figure S3) show the intra-monthly statistics in RnDR such as centrality (e.g., mean or median) and spread (e.g., the Inter Quartile Range, or IQR) within a month. The higher the IQR, the more variable the data of that month is. Therefore, a large IQR can be a sign of either a period in a year or from year to year when the environment conditions undergo considerable changes. In Yellowknife, for instance, the dose variations are generally greater during the cold months (e.g., Nov to Feb) and transitional months (e.g., Apr and May) than during the warm months. In Resolute, only the transitional months (May to July) experience a relatively large variation in RnDR.

SI-4: Principle Component Analysis

Assuming that no significant changes in geological and mineralogical conditions occurred during the measurement periods, meteorological variables then become the dominant factors influencing the RnDR levels at two study sites. These variables include atmospheric temperature, soil temperature and moisture, barometric pressure, precipitation (rainfall and snowfall), and snow depth on the ground. Note that soil temperature and moisture data are unavailable for this study.

A quick visual inspection of the variability of these available variables and their correlations with RnDRs can be helpful in interpreting the subsequent studies in seasonality and long-term trends.

This was accomplished using Principal Component Analysis (PCA), a statistical technique that can be employed to interpret and categorize features of multivariate data into reduced dimensional spaces known as Principal Components (PCs).

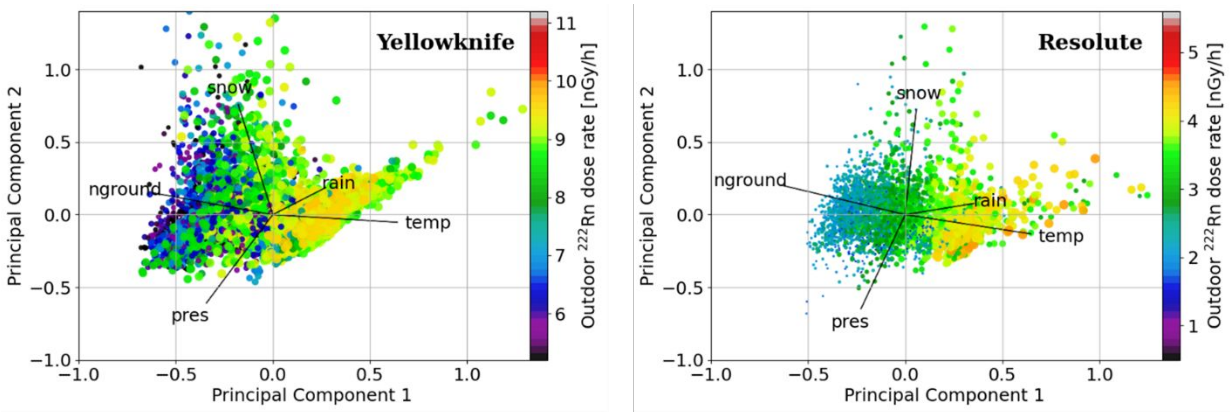

Figure S4: The principal component analysis biplots showing the meteorological variance and correlations, as well as the dose rate scatter data, in Yellowknife (left) and Resolute (right). The black lines present the loading vectors of the original axis of weather parameters. The magnitude of the dose rates is indicated by both the point size and the color pattern. Here “pres” stands for pressure, “temp” for temperature and “nground” for snow depth on ground.

In PCA analysis, all data sets were resampled on a daily basis to ensure consistent temporal resolution. Figure S4 shows the PCA results in two biplots, one for Yellowknife data and the other for Resolute data. In each plot, scattered points (i.e., scores) represent the projected weather variates in the new PC spaces, while black lines (i.e., loading vectors) illustrate the transformed axes of the original weather variables. The two newly constructed meteorological axes have the highest variance and are designated as PC1 and PC2 components. The cosine of the angle between two vectors indicates the correlation strength between variables.

In Figure S4, the PC1 and PC2 components respectively explain about 41.4% and 23.1% of the meteorological variance in Yellowknife, and around 34.8% and 22.7% variance in Resolute. In both cases, the primary variables contributing to PC1 variance are temperature and snow depth on the ground, while the PC2 variance is largely attributed to snowfall and pressure changes.

In Yellowknife, temperature is expectedly inversely correlated with snowfall and snow depth, while pressure is negatively correlated with precipitation. Resolute exhibits similar correlations, with the exception of the snowfall-temperature and snowfall-snow-depth relationships. Due to consistent cold weather throughout the year, snowfall can occur almost every day in Resolute, making it nearly independent of the other two variables.

Additionally, correlations of these weather variables with RnDRs can be qualitatively assessed by overlaying RnDR data into these biplots. To achieve this, each weather data point is resized and color-coded to indicate the magnitude of the paired RnDR. As such, the scatter plot incorporates both weather variability, demonstrated by the spatial distribution of data points, and RnDR magnitude, coded with colors and point size. The precipitation events cluster in space along rain and snow directions. However, RnDRs (indicated by either color or point size) do not exhibit large variations or clear trends along these directions, thus confirming the effectiveness of the data smoothing process. Among all directions, the overall RnDR variation is found to align more closely with directions of temperature and snow depth, both of which are also primary variables accounting for weather variance. As a result, temperature and snow depth are considered to be most relevant of all the available weather variables and hence are further considered in the main analyses.

SI-5: Annual Radon Dose Rate Cycle In Yellowknife

This supplementary section examines the annual evolution of RnDR in Yellowknife in relation to two weather variables: temperature and snow depth on the ground. In addition, similar distributions of ^{40}K dose rate and air concentrations of ^{210}Pb and ^{212}Pb were presented to illustrate the soil moisture attenuation effect, long-distance contribution and local soil gas emission.

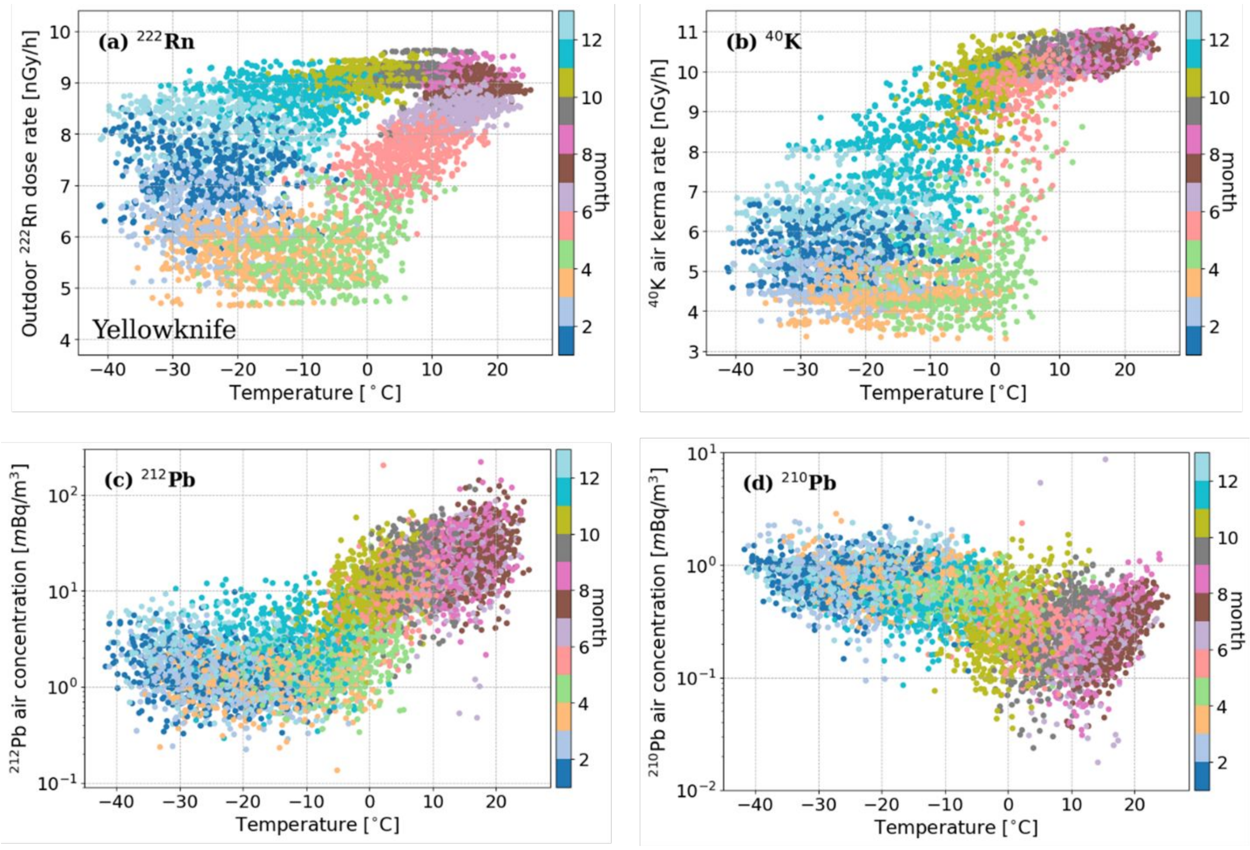

Figure S5: The annual cycles of the temperature dependent dose rates and air concentration measurements in Yellowknife. All data are daily averages. From January to December, the ^{222}Rn dose rate cycle follows a counter clockwise direction.

April is the month when Yellowknife begins to experience a reasonable number of days with temperatures above freezing (Figure S5) resulting from increased incoming solar irradiance (9 hours of sunshine daily on average). Early snow melting could commence in later March, and the entire melting process usually takes about a month (e.g., see Figures S6-a and -b). April clearly signals an upturn in ^{222}Rn and ^{40}K dose rates in a year cycle (Figures S5 and S6) due to reduced water load above ground and drying conditions. As compared to March, lower ^{210}Pb air concentration in April indicates a weakening long distance transportation contribution to Yellowknife in springtime (Figures 3-a and S5-d). Meanwhile, ^{212}Pb concentration in April is found to increase as compared to the previous few months (see Figures 3-a and S5-c), indicating an elevated radon gas emission from the soil.

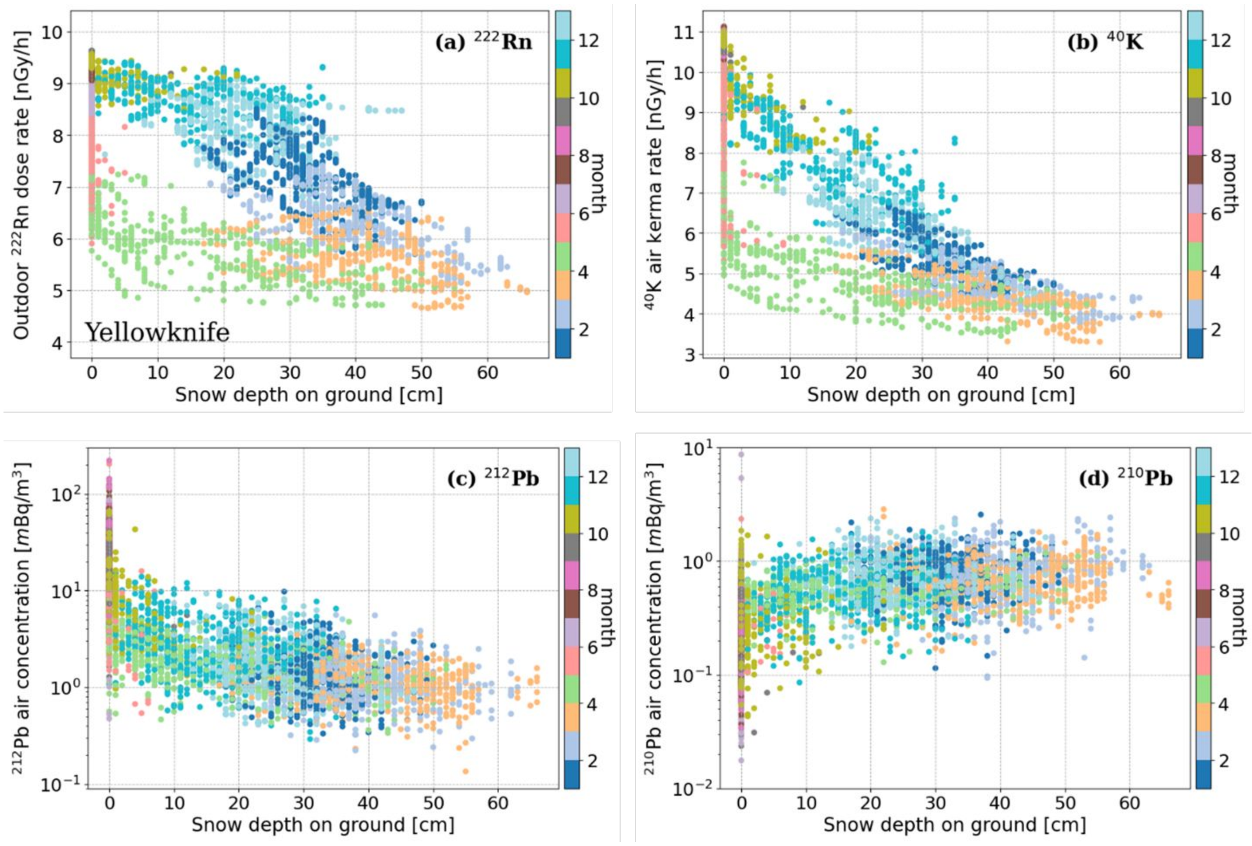

Figure S6: The annual cycles of the snow-depth dependent dose rates and air concentration measurements in Yellowknife. All data are daily averages. From January to December, the ^{222}Rn dose rate cycle runs clockwise.

As seen in Figures S6-a and -b, the dose rate variations in ^{222}Rn and ^{40}K from start of snow melting (around end of March) until only a few centimeters of snowpack remaining (around end of April) are at a modest level of approximately 1 nGy/h. During this period, the melted snow can either evaporate or runoff from the snow/ground surface or it can also accumulate in the remaining snowpack, coexist with the frozen ground surface or infiltrate into the thawing soil. Consequently, the actual water load above ground tends to change less significantly compared to the apparent change in snow depth. This can elucidate the gradual increase in both dose rates throughout most of April, as depicted in Figure S6. The melting of the last few centimeters of snowpack, which typically contain high amount of water, can lead to another dose rate increase of around 1 nGy/h in the ^{40}K and ^{222}Rn dose rates. The complete snow melting occurs around late April or early May, as indicated in Figure S6.

Yellowknife has a well-above freezing temperatures in May, with 11 to 12 hours of daily sunshine and a dry weather. In June, it becomes even warmer with an average temperature above 10 °C, but with slightly more precipitation. During these two months, the ground surface and shallow soil layer typically dry up quickly as soil thawing progresses, leading to increased probability of gamma transmission from the soil to above ground surface. The rapid rise in ^{40}K dose rate in May (Figure S5-b) confirms the drying condition in the shallow soil around the Yellowknife station during this season.

Furthermore, the drying condition in May also sets forth a stage of quick rising in RnDR, as seen in Figure S5-a. However, its increase rate is slower than that of the ^{40}K dose rate (see Figures 3-a and S5-b). It could have increased by a similar amount if all radon radionuclides were confined within the solid grain. However, in reality, a portion of radon in soil becomes mobile and can escape into the atmosphere. As indicated by the elevating ^{212}Pb air concentration during this period (see Figures 3-a and S5-c), radon gas release is also expected to increase over time. Since the observed RnDR is lower than what it would be if radon gas remained stationary in the soil, it is reasonable to speculate that the radon gas released into atmosphere becomes significantly diluted. Additionally, ^{210}Pb concentration gradually declines and attains at a relatively low level over the course of a year, indicating that the long-distance source is not contributing to the observed RnDR increase.

Gammas emitted from deeper soils have a smaller impact on above-ground exposure due to increased attenuation. Therefore, a similar change in soil conditions at deeper depths usually produces a smaller change in RnDR than the upper soil layer. This helps to explain the gradual increase in both ^{222}Rn and ^{40}K dose rates in June compared to the previous month, as shown in Figures 3-a and S5.

July is the warmest month in Yellowknife, and it also marks the start of the rainy season (July to October). As depicted in Figures 3-a and S5, ^{40}K dose rate peaks in July, implying that the active layer at this time has reached a soil depth of about 50 cm. Any soil condition changes further

below this depth have no significant effect on the above-ground dose rate. Over the next two months, ^{40}K dose rate shows a slight decline, most likely due to moisture accumulated in the soil during the rainy season. High soil moisture levels can also suppress the release of ^{220}Rn and ^{222}Rn gases to the atmosphere. ^{212}Pb concentration, as shown in Figures 3-a and S5-c, confirms this; its atmospheric concentration level reaches an inflection point in July, then declines steadily over the next two months.

In contrast, RnDR continues to increase during these three months, albeit only slightly. The observed increase must be attributed to mobile radon, as the dose rates from stationary radon are predicted to decline in a manner similar to the ^{40}K dose. Specifically, it is most likely due to reduced radon gas exhalation into the atmosphere and subsequent increase in soil ^{222}Rn concentration. The accumulated moisture appears to be one factor because it traps radon gas in the soil pore space, bringing the source and detector closer than when the gas is diluted in the atmosphere. Another possibility is that radon gas that originates below the intermediate depth can easily migrate upwards as the soil thaws and deepens over this period. This would also contribute favorably to the ground-level exposure. Further research with relevant data is required to confirm these sources and quantify their significance.

The first snowfall in Yellowknife typically occurs in late September or October, when the daily minimum temperature drops below the freezing (see Figure S5). As the ground surface freezes or snow accumulates, RnDR enters a prolonged suppression stage, which persists until the following year when snow melts. Both ^{40}K dose rate and ^{212}Pb air concentration are also suppressed during this period. In contrast, the ^{210}Pb air concentration starts to increase in October and peaks in either January or February. This essentially demonstrates the prevalent period of long-distance contribution in Yellowknife. Compared to the ^{40}K dose rate trend, RnDR exhibits a more gradual decrease over these months. Three non-stationary radon sources could be responsible for this slower decrease: accumulated radon gas in soil, radon trapped in snowpack and the long distance transportation.

As atmospheric temperature falls below zero degrees, active layer starts to freeze back downward from the surface. The frozen surface acts as a barrier, effectively preventing the release of radon gas into the atmosphere, as evidenced by the decreasing ^{212}Pb concentration. As a result, radon soil concentration increases within the unfrozen soil beneath the freezing front. It should be noted that radon gas originating from deep soil beyond the intermediate depth can migrate upward and contribute to this concentration increase. Meanwhile, a portion of radon gas that still manages to escape from the soil can become trapped in the snowpack on the ground. Radon in both scenarios tends to increase the above-ground exposure compared to when being released into the atmosphere otherwise. Furthermore, as evidenced by the ^{210}Pb measurements, the intensified long-distance transportation in winter could be a source. This study, however, does not have sufficient data to quantify these respective contributions.

It may take a couple of months for the freezing front to reach a depth of about 50 cm. From then on, all radon with the intermediate soil depth becomes nearly stationary and the geometrical relationship between the detector and source is fixed. As a result, the primary influence factors on RnDR are reduced to two factors: snow depth on the ground and long-distance transportation. If assuming the long-distance contribution is much smaller than the snow depth impact, the ^{222}Rn to ^{40}K dose rate (RnK) ratios are expected to covary and eventually approach a roughly constant ratio as the top 50 cm of soil freezes.

Table S1: monthly-averaged radon dose rates, radon to ^{40}K dose rate ratios and stationary fractions in Yellowknife.

	Jan	Feb	Mar	Apr	May	Jun	Jul	Aug	Sep	Oct	Nov	Dec
RnDR (nGy/h)	7.13	6.22	5.60	5.82	7.54	8.46	8.90	9.12	9.22	9.13	8.74	8.13
RnK ratio	1.34	1.34	1.29	1.14	0.78	0.81	0.84	0.87	0.90	0.93	1.11	1.28
Stationary fraction	1.00	1.00	0.96	0.85	0.58	0.60	0.63	0.65	0.67	0.69	0.83	0.95

Based on the data in Figure 3-a, RnK ratios were computed for each month and are presented in Table S1. It shows that January and February are the two months with constant ratios at 1.34. This implies that soil was refrozen at a depth below the intermediate level in the previous month December (RnK=1.28), whereas March (RnK=1.29) is likely to see a slightly thawing of the soil on the ground surface beneath the snow cover.

By assuming a fixed RnK ratio of 1.34 throughout the year, theoretical RnDRs for the scenario in which all soil radon is assumedly stationary can be calculated by multiplying this ratio with the ^{40}K dose rate of each month. These RnDRs can then be used to quantify the respective fractions of stationary and mobile radon components. Each month, the stationary fraction is calculated as the proportion of detected RnDR relative to the theoretical RnDR, as shown in Table S1. Because this definition assumes that the detected RnDR is solely from stationary radon, the calculated fraction is better referred to as an upper limit of stationary proportion (or 1-fraction is the lower limit of mobile proportion). These results suggest that mobile radon contributes the most to RnDR in May, when the shallow soil surface thaws and dries, at a 72% (0.42/0.58) ratio to the stationary portion.

SI-6: Annual Radon Dose Rate Cycle In Resolute

Different from Yellowknife, Resolute's RnDR annual cycle (see Figure 3-b) is divided into three stages: upturn, quick rising and suppression. It does not reach a plateau stage due to brief and cold summer. For these common stages, their onset times and durations in Resolute differ from Yellowknife's due to different weather conditions and possibly geological and terrain features.

Similar to the previous discussions, this supplemental section studies the dependence of RnDR, KDR and air concentrations on temperature and snow depth in Resolute, as seen in Figures S7 and S8.

It should be noted that the Resolute studies, as shown in Figures S7 and S8, used the weekly ^{210}Pb data from the CRMN station rather than the CTBT data. The change was due to the low efficiency and poor precision of the ^{210}Pb measurements obtained from the CTBT RASA detector

in Resolute. The RASA detector has a thick n-type contact that can considerably attenuate low energy gamma rays below 100 KeV.

In Figure S8, the snow depth dependence is hazy and dispersed. This is due to the polar desert climate in Resolute, where snowfall is limited yet can occur in practically any month of the year. As a result, the amount of snow on the ground can vary greatly both within a given month and across years. Additionally, snow drift can cause swift and abrupt changes. In this figure, snow depth in May ranges from about 30 cm to less than 10 cm. Because the mean temperature in May remains as low as -10 degrees, the observed snow depth range should primarily reflect variations within this month or across years rather than changes brought on by snowmelt.

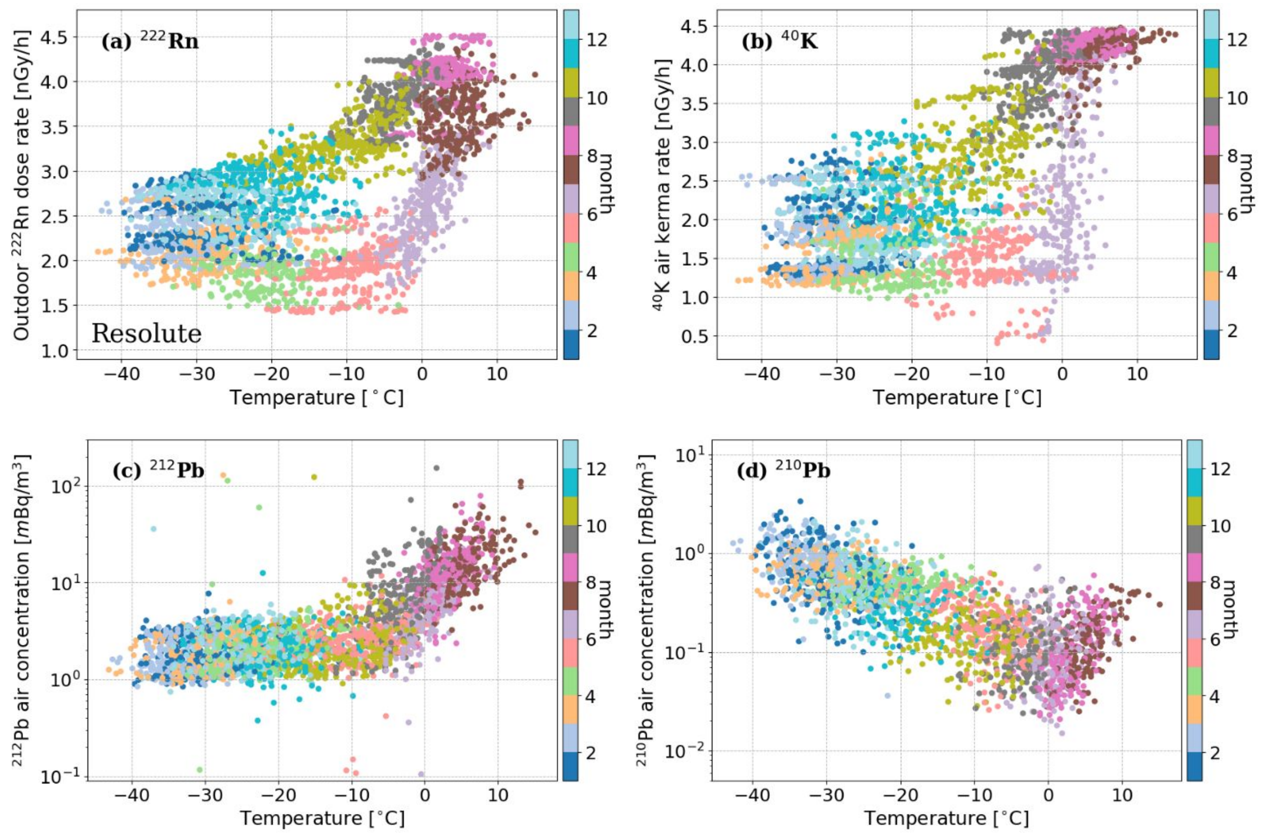

Figure S7: The annual cycles of the temperature dependent dose rates and air concentrations in Resolute. From January to December, the ^{222}Rn dose rate cycle follows a counter clockwise direction. Each data point here is the daily averaged result.

Alternatively, it is believed that June, the first month with a few days above zero degrees in Resolute, starts to see noticeable snowmelt due to the extended daylight hours and warmer weather (see Figures S7 and S8). As a result, a clear upturn can be found in June for both radon and ^{40}K dose rates, as well as the ^{212}Pb air concentration (see Figures 3-b, S7 and S8). Because a full snowmelt is also likely at the end of this month or in early July (see Figure S8), this month also sees rapid increases in these observations.

July is the warmest month in Resolute with daily highs around 7 ˚C and lows around 2 ˚C. It has about 4 cm of precipitation overall (rainfall and snowfall). The ^{40}K dose rate clusters at its maximum most of time in this month, as seen in Figure S7-b, whereas RnDR does not reach its peak until August (see Figure S7-a). Similar to the situation in Yellowknife, slow increase in

RnDR relative to ^{40}K dose rate is caused by a high radon gas release from soil to the atmosphere, as indicated by the ^{212}Pb concentration data (see Figures S7-c and 3-b) in this month.

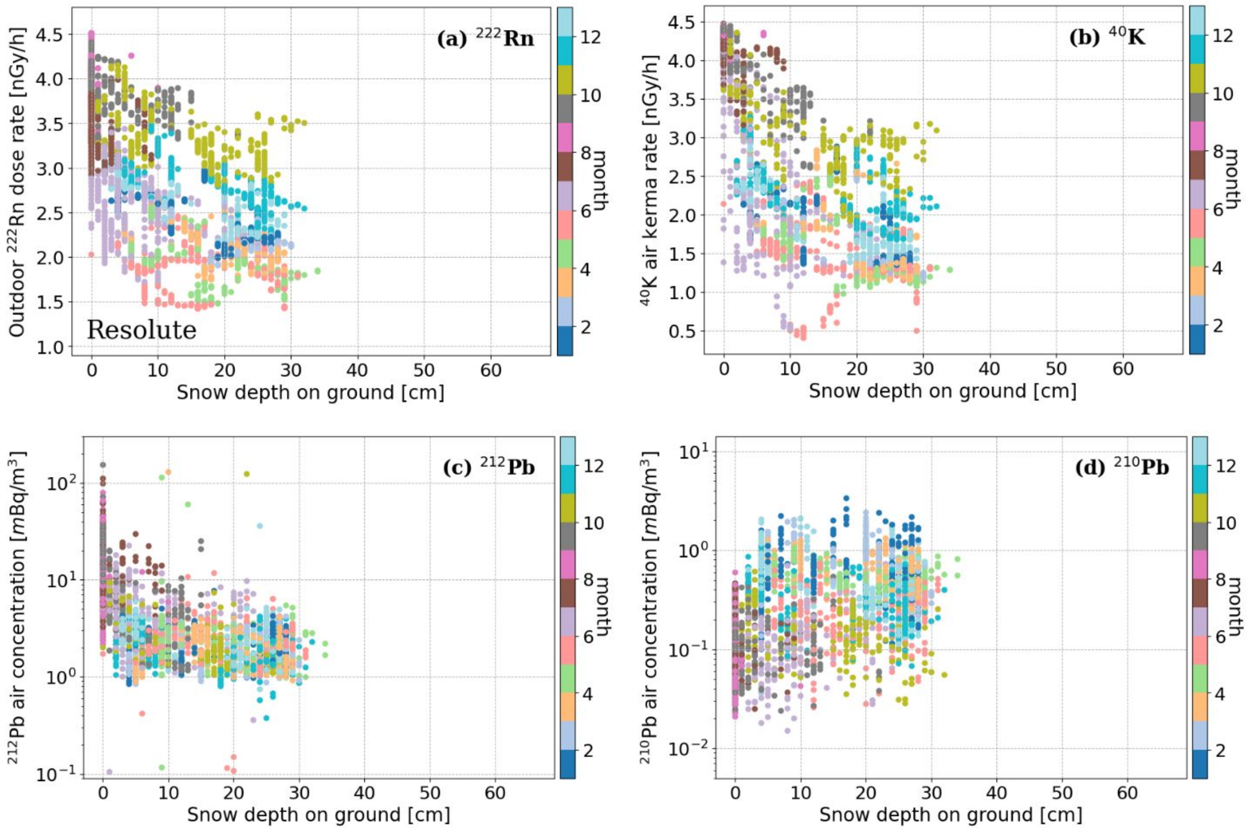

Figure S8: The annual cycles of the snow-depth dependent dose rates and air concentration measurements in Resolute. From January to December, the ^{222}Rn dose rate cycle runs clockwise. Each data point here is the daily averaged result.

As it comes to August, Resolute has daily highs of around 6 ˚C, but several days with lows below freezing. The total amount of precipitation in August is about 4-5 cm, slightly higher than in July. So, compared to July, the decrease in ^{212}Pb concentration in August is more likely caused by ground surface freezing during cold nights rather than by variations in soil moisture. Decreased ^{212}Pb air concentration indicates a high radon gas concentration in soil, which then explains the continuous increase in RnDR in August. On the other hand, this month's ^{40}K dose rate remains high, indicating that the soil active layer has likely descended close to or below the intermediate level.

From June to October, long-distance transportation contribution reaches and maintains its lowest level, according to the ^{210}Pb air concentration as show in Figure 3-b. so it is unlikely responsible for the rise in these observations (e.g., ^{40}K and ^{222}Rn dose rates) from June to August.

Table S2: monthly-averaged radon dose rates, radon to ^{40}K dose rate ratios and stationary fractions in Resolute.

	Jan	Feb	Mar	Apr	May	Jun	Jul	Aug	Sep	Oct	Nov	Dec
RnDR (nGy/h)	2.42	2.30	2.13	1.91	1.89	2.57	3.59	3.98	3.78	3.29	2.84	2.57
RnK ratio	1.39	1.41	1.36	1.28	1.35	1.12	0.87	0.94	0.98	1.13	1.24	1.33
Stationary fraction	0.99	1.00	0.97	0.91	0.96	0.80	0.62	0.67	0.70	0.80	0.88	0.94

Starting from September and lasting until May next year, RnDR enters to a long suppression stage, owing primarily to the accumulated snow on the ground. Likewise, as suggested by the decreasing ^{212}Pb concentration, ^{40}K dose rate and soil gas exhalation rate both gradually decline. The RnK ratios, as shown in Table S2, rise steadily from 0.98 in September to 1.33 in December before peaking at approximately 1.4 with slight variations from January to March. This basically reflects and follows the gradual soil freezing and soil radon stabilization processes. A nearly stable RnK ratio from January to March indicates that the soil frozen depth has dropped below the intermediate depth, or to its maximum if the active layer is shallower than the intermediate depth. In Resolute, the summer month July has the lowest stationary fraction (as shown in Table S2), indicating the highest radon gas release from the soil.

SI-7: Inter-quarterly correlation in Resolute's RnDR

The reason behind the rebounding point in Q4 of 2021 and Q1 of 2022 (Figure 6a-d of the main text) can be traced back to the reduced snowfall during Q4 of 2021, as indicated by the second-to-last snow data point in Figure 6d. Likewise, the uptick in RnDR in Q2 of 2022 (the last point in Figure 6b) can be linked to the same cause. Such inter-quarterly impacts are not uncommon in other years and can be illustrated by examining the covariance of the RnDR between quarters.

The analysis predicts a correlation coefficient of +0.867 between Q4 and Q1, +0.572 between Q4 and Q2, and +0.753 between Q1 and Q2. It should be emphasized that in these analysis the RnDR data points in Q1 or Q2 should be compared to the Q4 data from the preceding year.

These correlations are most likely caused by certain meteorological factors such as the SAWC that can build up over time and persist throughout the winter months. Resolute has a dry summer and the pre-winter rainfall that freezes can have long-lasting impact on RnDR. However, in contrast to the Yellowknife case, the impact of this form of SAWC is anticipated to be less significant than that of snowfall. In Resolute, the main snow season runs from September to November. As such, the amount of snowfall in Q4 usually has a significant and long-lasting impact on the RnDRs of the upcoming two quarters (the Q1 and Q2 of the new year). As shown in Figure 6d, the snowfall precipitation in Q4 accounts for the majority of the winter snowfall, as the difference in snow depth between Q4 and Q1, and Q1 and Q2 is small with respect to the snow depth in Q4. This explains the similarity in the variation pattern and inter-quarterly correlation of RnDR among these cold quarters.

SI-8: Long-term Trends on Warm Dry Days

As shown in Figures 5-y2 and 6-r2, temperature was found to be the only relevant variable in explaining the year-to-year RnDR variations of Q3 in Yellowknife and Resolute. In both cases, temperature was positively but marginally correlated with RnDR. In light of these findings, further study has been performed to re-estimate these relationships in a simplified scenario that is devoid of snow coverage and precipitation. This condition was achieved by selecting the Q3 data in days with a mean temperature above freezing (i.e., a warm day), and no precipitation and snow cover (i.e., a dry day). One purpose of this study is to check if fewer environmental factors could lead to a better constrained temperature-RnDR relationship. Additionally, it also aims to assess the effect of the data decomposition procedure by comparing the original and smoothed RnDRs on these particular days. However, it is acknowledged that with this selection the water content in soil may be still affected at a certain extent.

The selection ends up with about 60 ± 8 days of data in Yellowknife and 40 ± 13 days of data in Resolute. These results indicate that roughly one-third of the days in Yellowknife's Q3 is a rainy day because the majority of this quarter is above freezing. Differently in Resolute, the day selection is a combined result of temperature and precipitation conditions because certain days in Q3 have a chance of being wet (10% to 20%) or below freezing. In Figure S9, the year-by-year variations of temperature, the decomposed RnDR and the original RnDR on these selected days are displayed.

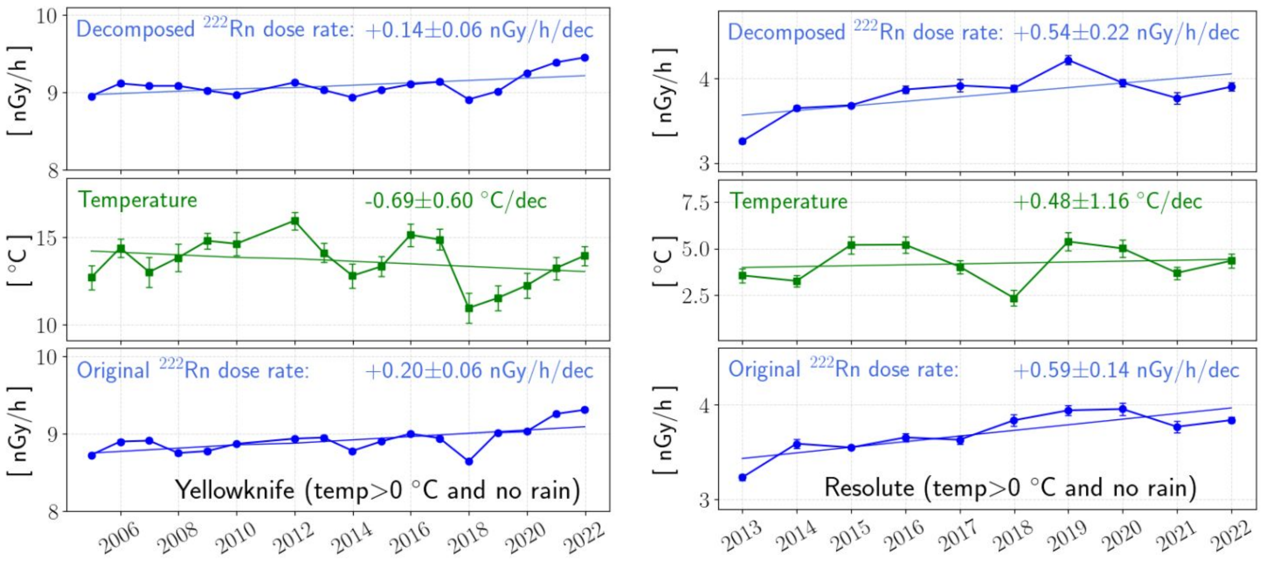

Figure S9: The annually average RnDRs and temperature in the warm dry days at the Yellowknife (left) and Resolute (right) stations. The linear regression trends are shown in lines and the slopes are given in the legends. The error bars shown in the dose rate plots are the standard errors of mean. The 2011 data was removed in Yellowknife plots due to incomplete dose rate measurements that year.

As shown in Figure S9, the smoothed RnDRs in Yellowknife are found to be strongly correlated with (r = .89) but are about 2% higher on average than the originals. In Resolute, the smoothed RnDRs are about 2.5% higher than the originals while both are positively correlated with an r-value of 0.91. Since these data were collected on days without precipitations, no significant precipitation effects are expected; however, post-precipitation shielding effect is anticipated. The compensation for the shielding effect during the decomposition process should therefore account

for the relatively higher RnDRs seen in the smoothed data compared to the originals at both stations. The compensation is at a level of 2% to 2.5% on average.

As to the long-term trend, a rising tendency is seen in all RnDRs at both stations. The rise in Yellowknife was primarily driven by data from the recent years. Its smoothed RnDRs are found to have a rising rate 30% slower than that of the originals. In Resolute, the smoothed RnDRs exhibits a rising trend that is approximately 8% lower than the original data, while both show a widely distributed rising pattern over the majority of years. These results suggest that at both stations the smoothed RnDR tends to have a weaker rising trend than originals, even though it can be argued that both rates are statistically comparable. This is likely attributed to the data centralizing and smearing effects during the data smoothing process. Furthermore, these effects are likely amplified as precipitation is more abundant, either in terms of quantity or frequency. This probably explains why the long-term trend of the smoothed data in Yellowknife is much weaker than its original as compared to the difference between Resolute's smoothed and original RnDRs.

In Yellowknife, the temperature on these selected days has a slightly cooling tendency but not to a statistically significant extent due to the large year-to-year fluctuation. It was positively correlated with the smoothed RnDRs with an r-value of 0.204 and a β coefficient of 0.023 nGy/h per °C. Here the r-value and the β coefficient are approximately half of those obtained with all of the Q3 data. In Resolute, the temperature on these selected days does not deviate from its zero trend. It was positively correlated with the smoothed RnDRs with an r-value of 0.402 and a β coefficient of 0.101 nGy/h per °C. Both values are less than their respective counterparts obtained with the entire Q3 data, which are 0.595 in r-value and 0.115 nGy/h per °C in β coefficient, respectively. In both cases, the temperature-RnDR associations were actually not improved but became weaker on these specific days compared to the whole Q3. One interpretation for this is that other environmental factors, like soil moisture content or the

distribution of radon gas, probably become more dynamic and variable on these non-precipitation days.

SI-9: Trend and correlation results

Table S3 summarizes all of the trends and correlations from both the yearly and quarterly regression analyses. Here the trends were obtained through linear regression analyses, while correlations are the Pearson correlation coefficients from univariate regression analyses.

SI-10: Feature Relevance With Multivariate Linear Regression Analyses

This section contains all feature relevance results obtained from multivariate linear regression analyses. As seen in Tables S4 and S5, ranked variables of relevance are presented, together with adj_R^2 contributions and relationship coefficient βs.

In the regression analysis, for a specific feature, its importance was assessed based upon the pre-existing variables in the model, which may have certain degree of collinearity with the feature being estimated. In such scenarios, this feature may appear to be less important or even lack significance. The univariate-based correlation outcomes in Table S3 of the main text can also be used to check the importance of (or the proportion of the variance explained) a specific variable.

Table S3: Long-term trends and correlations determined with Yellowknife (a-b) and Resolute (c-d) data. The long-term trends were determined using a simple linear regression model. The r^2 values are the coefficients of determination. The symbols "-" indicate no analyses were performed in these instances. The numbers in gray represent values that have no statistical significance (i.e., $p > .32$). In the correlation coefficients (rs) results, the numbers in gray are these with r values less than 0.3. Additional descriptions of these analyses can be found in online methods.

**Yellowknife long-term trends (a) and correlations (b)**							
		^{222}Rn dose rate (nGy/h/dec) [*p*, r^2]	Temperature (°C/dec) [*p*, r^2]	Snow depth (cm/dec) [*p*, r^2]	^{40}K dose rate (nGy/h/dec) [*p*, r^2]	^{212}Pb air conc. (mBq/m^3/dec) [*p*, r^2]	^{210}Pb air conc. (mBq/m^3/dec) [*p*, r^2]
(a) Long-term	Q1	**0.670 ± 0.151**<br>[.000, .550]	**-1.505 ± 1.029**<br>[.163, .118]	-3.197 ± 3.289<br>[.346, .056]	**0.417 ± 0.188**<br>[.041, .235]	**0.319 ± 0.115**<br>[.013, .327]	**-0.085 ± 0.066**<br>[.217, .093]
	Q2	**0.177 ± 0.107**<br>[.117, .146]	**-1.059 ± 0.583**<br>[.088, .171]	1.168 ± 1.201<br>[.345, .056]	0.105 ± 0.177<br>[.559, .022]	0.184 ± 1.405<br>[.898, .001]	**-0.045 ± 0.021**<br>[.047, .225]
	Q3	**0.136 ± 0.062**<br>[.044, .243]	0.037 ± 0.592<br>[.951, .000]	-	**0.086 ± 0.061**<br>[.177, .118]	2.176 ± 2.802<br>[.449, .039]	0.008 ± 0.022<br>[.732, .008]
	Q4	**0.468 ±0.127**	**-1.132 ± 0.796**	0.657 ± 1.967	**0.274 ± 0.199**	**1.001 ± 0.560**	-0.023 ± 0.031

		[.002, .477]	[.176, .119]	[.743, .007]	[.188, .113]	[.094, .176]	[.483, .033]
	Annual	**0.348 ± 0.060** [.000, .692]	**-1.150 ± 0.467** [.026, .288]	-0.231 ± 0.898 [.801, .004]	-	-	-
(b) Correlations with RnDR (rs)			Temperature	Snow depth	^{40}K dose rate	^{212}Pb air conc.	^{210}Pb air conc.
		Q1	**-0.399**	**-0.542**	**+0.882**	**+0.700**	-0.137
		Q2	+0.204	**-0.418**	**+0.842**	**+0.352**	**-0.321**
		Q3	**+0.395**	-	**+0.920**	+0.073	+0.137
		Q4	**-0.514**	-0.298	**+0.815**	**+0.499**	**-0.474**
		Annual	**-0.374**	-0.268	-	-	-
**Resolute long-term trends (c) and correlations (d)**							
		^{222}Rn dose rate (nGy/h/dec) [*p*, r^2]	Temperature (°C/dec) [*p*, r^2]	Snow depth (cm/dec) [*p*, r^2]	^{40}K dose rate (nGy/h/dec) [*p*, r^2]	^{212}Pb air conc. (mBq/m^3/dec) [*p*, r^2]	^{210}Pb air conc. (mBq/m^3/dec) [*p*, r^2]
(c) Long-term trends	Q1	**-0.543 ± 0.219** [.038, .435]	0.358 ± 1.506 [.818, .007]	7.674 ± 8.244 [.379, .098]	**-0.921 ± 0.417** [.058, .379]	**0.782 ± 0.709** [.302, .132]	**-0.282 ± 0.211** [.219, .182]
	Q2	**-0.434 ± 0.234** [.100, .301]	0.455 ± 0.671 [.516, .054]	-0.401 ± 6.885 [.955, .000]	**-1.270 ± 0.334** [.005, .644]	-0.535 ± 1.061 [.628, .031]	**-0.111 ± 0.049** [.055, .386]
	Q3	**0.615 ± 0.186** [.011, .578]	**1.523 ± 1.377** [.301, .133]	-0.175 ± 2.160 [.937, .001]	**0.304 ± 0.168** [.108, .290]	**4.982 ± 2.740** [.106, .292]	-
	Q4	-0.031 ±0.223 [.893, .002]	**3.596 ± 1.876** [.092, .315]	0.657 ± 1.967 [.743, .007]	-0.338 ± 0.407 [.401, .079]	0.256 ± 0.928 [.790, .009]	**-0.319 ± 0.098** [.012, .570]
	Annual	-0.094 ± 0.128 [.485, .063]	**1.484 ± 0.794** [.099, .304]	3.106 ± 5.626 [.596, .037]	-	-	-
(d) Correlations with RnDR (rs)			Temperature	Snow depth	^{40}K dose rate	^{212}Pb air conc.	^{210}Pb air conc.
		Q1	-0.160	**-0.584**	**+0.923**	+0.212	**+0.581**
		Q2	-0.256	-0.093	**+0.853**	**+0.596**	+0.247
		Q3	**+0.595**	+0.059	**+0.331**	**+0.533**	+0.210
		Q4	-0.187	**-0.708**	**+0.825**	**+0.495**	**+0.581**
		Annual	-0.043	**-0.446**	-	-	-

It is also worth to mention that, variables in both the univariate and the multivariate analysis are not necessarily in a cause-and-effect relationship with RnDR, which should be assessed alternatively and independently based on common understandings and interpretations.

Table S4: feature relevance with Yellowknife data. Variables with an adj_R^2 less than 0.02 are shown in gray.

**Yellowknife feature relevance**			
		**(a)** with ^{40}K dose rate	**(b)** without ^{40}K dose rate
Ranking: Feature [Δadj_R^2, β]	Q1	**1st:** ^{40}K dose rate [0.759, 0.934 ± 0.118]<br>**2nd:** ^{210}Pb conc. [0.029, -0.650 ± 0.365 nGy/h per mBq/m^3]	**1st :** ^{212}Pb conc. [0.393, 0.630 ± 0.270 nGy/h per mBq/m^3]<br>**2nd :** snow depth [0.090, -0.032 ± 0.011 nGy/h per cm/day]<br>**3rd :** temperature [0.129, -0.087 ± 0.033 nGy/h per °C]<br>4th : ^{210}Pb conc. [0.019, -0.637 ± 0.485 nGy/h per mBq/m^3]
	Q2	**1st :** ^{40}K dose rate [0.541, 0.414 ± 0.116]<br>2nd : ^{212}Pb conc. [0.004, 0.024 ± 0.016 nGy/h per mBq/m^3]<br>3rd : rain_cq [0.008, 0.183 ± 0.165 nGy/h per mm/day]	**1st :** ^{212}Pb conc. [0.133, 0.052 ± 0.020 nGy/h per mBq/m^3]<br>**2nd :** rain_cq [0.066, 0.353 ± 0.213 nGy/h per mm/day]<br>3rd : ^{210}Pb conc. [0.013, -1.203 ± 1.081 nGy/h per mBq/m^3]
	Q3	**1st :** ^{40}K dose rate [0.835, 1.059 ± 0.104]<br>**2nd :** rain_cq [0.021, 0.069 ± 0.033 nGy/h per mm/day]<br>3rd : ^{210}Pb conc. [0.010, 0.422 ± 0.302 nGy/h per mBq/m^3]	**1st :** temperature [0.100, 0.047 ± 0.028 nGy/h per °C]
	Q4	**1st:** ^{40}K dose rate [0.636, 0.851 ± 0.153]	**1st :** rain_pq [0.229, -0.143 ± 0.171 nGy/h per mm/day]

		2nd: rain_pq [0.100, -0.356 ± 0.113 nGy/h per mm/day]<br>3rd: snow depth [0.075, 0.044 ± 0.018 nGy/h per cm/day]<br>4th: temperature [0.016, -0.040 ± 0.023 nGy/h per °C]<br>5th: ^{212}Pb conc. [0.010, -0.068 ± 0.053 nGy/h per mBq/m^3]	2nd: temperature [0.109, -0.057 ± 0.039 nGy/h per °C]<br>3rd: ^{210}Pb conc. [0.114, -2.584 ± 1.010 nGy/h per mBq/m^3]<br>4th: ^{212}Pb conc. [0.076, 0.107 ± 0.061 nGy/h per mBq/m^3]

Table S5: feature relevance with Resolute data. Variables with an adj_R^2 less than 0.02 are shown in gray.

Resolute feature relevance			
		**(a)** with ^{40}K dose rate	**(b)** without ^{40}K dose rate
Ranking: Feature [Δadj_R^2, β]	Q1	1st: ^{40}K dose rate [0.844, 0.511 ± 0.070]<br>2nd: temperature [0.011, -0.031 ± 0.024 nGy/h per °C]	1st: snow depth [0.258, -0.014 ± 0.010 nGy/h per cm/day]<br>2nd: ^{210}Pb conc. [0.088, 0.520 ± 0.361 nGy/h per mBq/m^3]
	Q2	1st: ^{40}K dose rate [0.476, 0.365 ± 0.120]	1st: ^{212}Pb conc. [0.076, 0.174 ± 0.089 nGy/h per mBq/m^3]<br>2nd: temperature [0.072, -0.167 ± 0.130 nGy/h per °C]<br>3rd: rain_cq [0.006, 0.788 ± 0.767 nGy/h per mm/day]
	Q3	1st: temperature [0.274, 0.115 ± 0.055 nGy/h per °C]	
	Q4	1st: ^{40}K dose rate [0.673, 1.004 ± 0.219]<br>2nd: ^{212}Pb conc. [0.045, -0.203 ± 0.069 nGy/h per mBq/m^3]<br>3rd: ^{210}Pb conc. [0.056, -0.782 ± 0.410 nGy/h per mBq/m^3]<br>4th: rain_pq [0.058, -0.165 ± 0.094 nGy/h per mm/day]	1st: snow depth [0.439, -0.011 ± 0.006 nGy/h per cm/day]<br>2nd: ^{210}Pb conc. [0.027, 0.508 ± 0.430 nGy/h per mBq/m^3]

SI-11: Other possible influencing factors

In this study, certain assumptions have been made to derive and interpret the results, including a steady geodynamic state throughout the study period. However, it notes that other conditions, if occur, can also affect the results reported in this work. For example, mining activities, changes in land caused by permafrost degradation, or geodynamic events can lead to abrupt/gradual shift in radon gas concentrations or dose rates. The following provides a general description of the geodynamics of Yellowknife and Resolute areas to help understand the chances and potential influences.

The Northwest and Nunavut regions of Canada, which include Yellowknife and Resolute, are primarily situated with the Canadian shield. Geodynamically, these regions are characterized by stable cratonic blocks, little tectonic activity and low seismicity. Despite being generally considered as stable, the Canada shield is not completely devoid of tectonic activity. Rifts, faults, and fractures have been formed as a result of episodes of tectonic activity in the past. These events were however not often or intense. Seismic events occur occasionally but are typically of low magnitude and infrequent. These events are mainly connected to the reactivation of ancient faults or stress release within the crust.

Overall, there may be some geodynamic events occurring in both regions within the study period, which however requires additional data/measurements to identify and characterize. Upon the availability of these relevant data, study on their impacts on radon measurements can be conducted.

Reference

Press, W.H. and Teukolsky, S. A., 1990. Savitzky-Golay Smoothing Filters, Computers in Physics 4, 669 (1990). doi: 10.1063/1.4822961.

Savitzky, A. and Golay, M., 1964. Soothing and differentiation of data by simplified least squares procedures, Anal. Chem., vol. 36, pp. 1627–1639

Tabachnick, B.G. and Fidell, L.S. (2007). Using multivariate statistics (5th ed.). Allyn & Bacon/Pearson Education.
